# The use of hybrid operating rooms in neurosurgery, advantages, disadvantages, and future perspectives: a systematic review

**DOI:** 10.1007/s00701-023-05756-7

**Published:** 2023-08-16

**Authors:** Maria Gharios, Victor Gabriel El-Hajj, Henrik Frisk, Marcus Ohlsson, Artur Omar, Erik Edström, Adrian Elmi-Terander

**Affiliations:** 1 Department of Clinical Neuroscience, Karolinska Institutet, Stockholm, Sweden; 2 Department of Neurosurgery, Karolinska University Hospital, Eugeniavägen 6, 4Th Floor, Solna, 17164 Stockholm, Sweden; 3 Department of Neuroradiology, Karolinska University Hospital, Stockholm, Sweden; 4 Department of Medical Radiation Physics and Nuclear Medicine, Karolinska University Hospital, Stockholm, Sweden; 5 Department of Surgical Sciences, Uppsala University, Uppsala, Sweden

**Keywords:** Hybrid operating rooms, Surgery, Spine, CBCT, Neurosurgery, Neurointerventional procedures

## Abstract

**Background:**

Hybrid operating rooms (hybrid-ORs) combine the functionalities of a conventional surgical theater with the advanced imaging technologies of a radiological suite. Hybrid-ORs are usually equipped with CBCT devices providing both 2D and 3D imaging capability that can be used for both interventional radiology and image guided surgical applications. Across all fields of surgery, the use of hybrid-ORs is gaining in traction, and neurosurgery is no exception. We hence aimed to comprehensively review the use of hybrid-ORs, the associated advantages, and disadvantages specific to the field of neurosurgery.

**Materials and methods:**

Electronic databases were searched for all studies on hybrid-ORs from inception to May 2022. Findings of matching studies were pooled to strengthen the current body of evidence.

**Results:**

Seventy-four studies were included in this review. Hybrid-ORs were mainly used in endovascular surgery (*n* = 41) and spine surgery (*n* = 33). Navigation systems were the most common additional technology employed along with the CBCT systems in the hybrid-ORs. Reported advantages of hybrid-ORs included immediate assessment of outcomes, reduced surgical revision rate, and the ability to perform combined open and endovascular procedures, among others. Concerns about increased radiation exposure and procedural time were some of the limitations mentioned.

**Conclusion:**

In the field of neurosurgery, the use of hybrid-ORs for different applications is increasing. Hybrid-ORs provide preprocedure, intraprocedure, and end-of-procedure imaging capabilities, thereby increasing surgical precision, and reducing the need for postoperative imaging and correction surgeries. Despite these advantages, radiation exposure to patient and staff is an important concern.

**Supplementary Information:**

The online version contains supplementary material available at 10.1007/s00701-023-05756-7.

## Introduction

A hybrid operating room (hybrid-OR) consists of a conventional surgical theater equipped with advanced imaging systems. The concept of a hybrid-OR was initially described for cardiovascular procedures by Barstad et al. [3]. At first, imaging was performed using mobile equipment, while ceiling- and floor-mounted imaging equipment were developed later. The integration of imaging equipment in the OR requires space and careful positioning to maximize the utility of the hardware while avoiding disruption of the surgical workflow and allowing rapid conversion to open procedures if needed [76]. Consequently, hybrid-ORs may be easier to install de novo in a new building as conversion of existing ORs may be very costly and still suffers from lack of space. Hence, both budget and space restrictions impact the installation and adoption of hybrid-ORs in modern surgical practice. Nonetheless, the past decade has witnessed an increase in the use of hybrid-ORs, in terms of both the number of procedures performed as well as the number of surgical specialties employing them [68]. In fact, hybrid-ORs are currently being used in cardiothoracic, vascular, orthopedic, otolaryngology/cervicofacial surgery, oral and maxillofacial surgeries, urology, as well as neurosurgery [68]. According to a recent scoping review of the literature, most of the research on hybrid-ORs has been conducted within the field of thoracic surgery, reporting the performance of procedures such as tumor resection or ablation, lung biopsy, and image-guided video-assisted thoracoscopic surgery [68].

Intraoperative imaging holds the promise of improved patient care. The ability to visualize anatomical and surgical information in detail can increase surgical accuracy and hence patient safety. With intraoperative imaging, neurosurgeons can acquire updated, and real time, images of the patient in the correct surgical positioning, rather than having to rely on preoperative scans performed in standard positions. In the hybrid-OR, 3D imaging can assist neurosurgeons at different stages of a surgery: preoperatively to visualize the pathology of interest and plan the surgical approach, intraoperatively to provide updated information and assess possible adverse events, and postoperatively for the immediate evaluation of surgical results to allow for corrections when needed. Hybrid-ORs also provide a space where neurosurgeons and interventionalists can collaborate to offer novel treatment options. However, repeated intraoperative imaging and multidisciplinary approaches may interrupt the surgical workflow and unnecessarily complicate procedures [15]. Another major concern is the potential to increase radiation exposure to both patients and staff [11]. It is therefore crucial to determine what procedures will benefit from the use of a hybrid-OR to the extent that the possible disadvantages are outweighed. Based on the latest consensus definition, a hybrid-OR is a surgical room equipped with a coordinate-based imaging system such as CT, MRI, or CBCT, in combination with auxiliary imaging techniques such as fluorescence and ultrasound. In neurosurgery, cone-beam computed tomography (CBCT) has been the most commonly used intraoperative 3D imaging modality [68]. A CBCT system consists of an x-ray tube and image detector that rotates around the patient, capturing a series of (2D) projections using a cone-shaped x-ray beam. The acquired image data are processed using a cone-beam reconstruction algorithm to generate 3D data of the patient anatomy. In contrast to conventional CT imaging, an extended volume can be captured with a single rotation of the CBCT, thereby reducing the scan time and minimizing movement artifacts [40, 59]. This systematic review provides an overview of the applications of hybrid-ORs utilizing fixed CBCT-based radiological systems in the field of neurosurgery, with a focus on their advantages and disadvantages, including increased radiation exposure. Finally, future perspectives based on the gathered information will be discussed.

## Methods

This systematic review is reported in accordance with both the Preferred Reporting Items for Systematic Reviews and Meta-Analyses (PRISMA) [58] as well as the Assessing the Methodological Quality of Systematic Reviews (AMSTAR) guidelines [65]. The related 2020-PRISMA and AMSTAR 2 checklists are provided as supplementary material (Supplementary file 1 and 2). The review protocol was registered within the International Prospective Register of Systematic Reviews (PROSPERO; *date of registration* 13 July, 2022*)*. The record was consistently updated in the event of major changes to the workflow or study design.

### Databases and search strategy

A query combining the keywords “hybrid,” “operating room,” “CBCT,” and “neurosurgery” was used to search in PubMed, Web of Science, and Embase (Supplementary file 3).

### Study selection

**Fig. 1:**
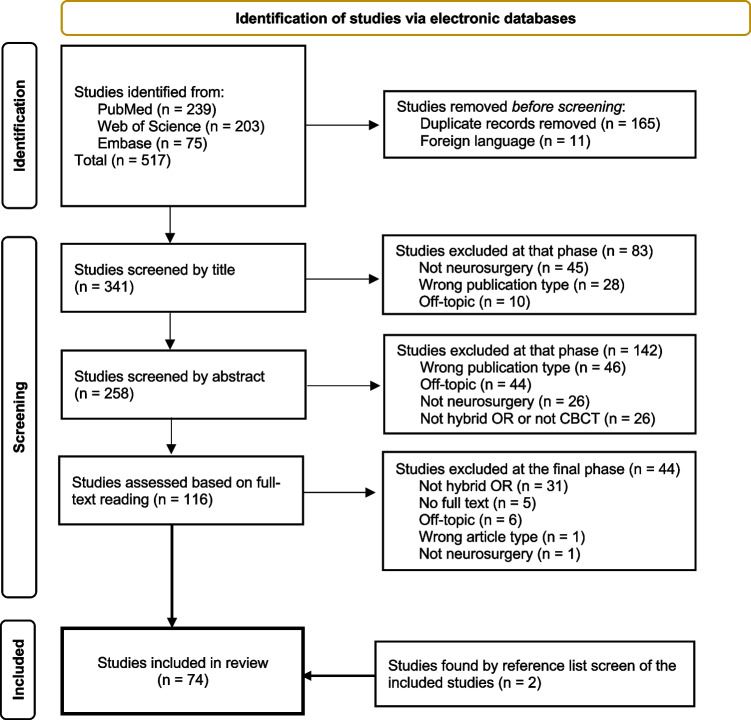
PRISMA 2020 flow diagram

As of May of 2022, the database search yielded a total of 517 papers. Once retrieved onto Rayyan [57] and after deduplication, 341 records were screened by two independent and blinded reviewers (M.G. and V.G.E.), first by title, and then by abstract. The remaining articles were gathered in full text and assessed by three independent and blinded reviewers (M.G., A.E.T., E.E.). Conflicts were resolved through team discussion and subsequent unanimous decision. Applying the eligibility criteria (Table 1), the screening process resulted in 270 exclusions, leaving a total of 74 studies, as presented in the PRISMA flowchart (Fig. 1). Finally, two articles were added after a reference list screening of the included articles was performed.

**Table 1:** Inclusion and exclusion criteria applied during the article selection process

Criteria	Inclusion	Exclusion
Study type	Original empirical studies and technical reports	Reviews, letters, conference abstracts, and case reports
Study timing	n/a	n/a
Study language	English	Non-English studies
Population	n/a	n/a
Device used	• Hybrid-OR • Stationary, ceiling-, or floor-mounted x-ray imaging system • CBCT modality	• Mobile radiological x-ray imaging system (ex: O-arm) • Non-CBCT modality
Intervention	Neurosurgery and spine surgery	n/a
Comparator	n/a	n/a
Outcome	Areas of use, advantages and disadvantages, radiation exposure	n/a

### Data synthesis and risk of bias assessment

Due to the great heterogeneity with respect to methodology, design, and outcomes reported among the studies, neither pooling nor meta-analysis of data was possible. We hence opted for a qualitative and descriptive synthesis of the available literature. Studies with overlapping cohorts were delt with in a manner such as the same data was never regarded twice during synthesis. In addition, a thorough appraisal of the risk of bias in the studies included was performed using the Newcastle–Ottawa scale (NOS) [73] for non-randomized studies and the Cochrane Risk of Bias Tool for the randomized controlled trial. A similar methodology was adopted in previous works [18, 19, 30, 70].

## Results

### Study characteristics

Among the 74 studies included, 59 (80%) were published during the last 5 years (between 2017 and 2022), and 15 (20%) between 2006 and 2016. Overall, the studies originated from 14 different countries, with Europe (50%) and Asia (47%) contributing equally. The remainder of the studies (3%) was from the USA. Only one randomized control trial (1.5%) was identified. In total, there were 26 studies (35%) that presented control or comparison groups (Supplementary file 4). While case reports were excluded from the main analysis, an overview of the latest and most intriguing ones is presented as supplementary (Supplementary file 4).

### Risk of bias assessment

The scores for cohort studies ranged from 3 to 9 of a possible total of 9, with a median of 6. The two case–control studies both scored 8 out of 8. The randomized controlled study was judged to be of “fair quality” based on the Cochrane risk of bias tool (Supplementary file 5).

### Procedures performed in the hybrid-OR

Hybrid-ORs were mainly employed in two areas of neurosurgery: cerebrovascular including endovascular surgery (*n* = 41) and spine (*n* = 33). Other areas were represented in eight studies. Several of the studies had addressed more than one area of application, and the presented numbers are non-exclusive. A wide array of procedures was carried out within the hybrid-OR, the most common ones were the treatment of different cerebrovascular pathologies, such as aneurysms (*n* = 26), AVMs (*n* = 20), and AVFs (*n* = 12). Of these, 25 studies reported on combined open and endovascular treatments in the hybrid-OR. Spinal instrumentation procedures were also frequently performed in hybrid-ORs (*n* = 27). Other procedures included evacuation of brain hemorrhages (*n* = 6), carotid endarterectomy and stenting (*n* = 6), and resection of skull base tumors (*n* = 2). Two studies reported the use of the hybrid-OR only for imaging purposes, while details regarding the procedures were not available in three studies.

### Intraoperative imaging systems

The CBCT systems used were mainly the Allura (Philips Healthcare, Best, The Netherlands; *n* = 37), Artis (Siemens AG, Forchheim, Germany; *n* = 27), UNIQ (Philips Healthcare, Best, The Netherlands; *n* = 1), INFX-8000 V (Canon Medical Systems Corp., Tochigi, Japan; *n* = 1), Discovery Image-Guided System 730 (GE Healthcare, Milwaukee, Wisconsin, USA; *n* = 1), and a system manufactured by Toshiba (Toshiba, Tokyo, Japan; *n* = 1). In six studies, the systems used were undisclosed.

### Use of the intraoperative imaging

#### Preoperative imaging in the OR

The use of CBCT for preoperative imaging at the start of a procedure was mentioned in 33 studies. Preoperative imaging was used to acquire the necessary imaging data for intraoperative navigation systems.

#### Imaging during the procedure

Fifty-seven studies reported the use of CBCT during the procedure, where three separate applications were found: intraoperative imaging, interventional guidance, and neuronavigation.

Intraoperative imaging (*n* = 22) refers to the acquisition of 3D imaging data during the surgery to obtain updated information. Interventional guidance (*n* = 33) involves live imaging during an endovascular procedure. Neuronavigation (*n* = 31) combines 3D imaging data with real-time positional tracking of the patient and surgical instruments. This provides accuracy and allows precise procedures to be performed even when the anatomical structures are unseen such as in minimally invasive procedures. These images were generally obtained at the start of a procedure. However, the hybrid-OR provided the possibility to update the 3D image set used by the navigation software. In two studies, new images were obtained during the procedure.

#### Imaging at the end of the procedure

The use of CBCT for end-of-procedure (EOP) imaging was reported in 56 studies. In these cases, EOP imaging was mainly used to assess surgical result and evaluate the need for adjustments. Three studies stated that EOP imaging in the hybrid-OR was as effective as conventional postoperative imaging for evaluation of surgical results [9, 44, 52]. Three studies on spinal surgery indicated that EOP scans were particularly useful for the detection of cases requiring revision. In cerebrovascular procedures, 23 studies showed that EOP imaging allowed the detection of parent artery occlusion, residual perfusion of a pathological vessel, or remnants of vascular anomalies such as aneurysms or AVMs. These findings lead to a prolongation of the surgical or interventional procedure as stated in 20 of these studies. In tumor surgery, EOP imaging was mainly used to identify cases where more extensive resection was needed.

### Additional technologies used in the hybrid-OR

Forty-four studies reported the use of additional technologies in the hybrid-OR. The use of surgical navigation or surgical robots in spine surgery was reported in 23 studies, the use of indocyanine green video angiography (ICGV) and intraoperative angiography in cerebrovascular surgery in 22 studies, and other technologies including surgical navigation and ultrasound in cranial surgery were reported in four studies.

#### Cerebrovascular

In cerebrovascular surgery, the most commonly used additional medical technology in the hybrid-OR was intraoperative near-infrared indocyanine green video angiography (ICGV) (*n* = 16), a tool for cerebral blood flow assessment. It was performed at the end of the procedure in 13 studies to evaluate the treatment result. A discordance between ICGV investigation and subsequent control angiography was reported in 12 of these studies, with the intraoperative angiography identifying additional residual aneurysms/AVM/fistulas that were not detected by ICGV. Only one study reported complete agreement between ICGV and intraoperative angiography [66]. Another technology employed inside the hybrid-OR was ultrasound (*n* = 10). Eight studies used Doppler sonography along with ICGV as routine vascular monitoring at the end of the procedure. Eight studies reported the use of neuronavigation. Brainlab navigation (Brainlab, Munich, Germany) was used in five and the XperGuide (Philips Medical Systems, Best, The Netherlands) in one study. One study reported the use of augmented reality for optimal planning of minimally invasive craniotomy [24]. The remaining two studies did not specify which navigation platform they employed. Intraoperative MRI was performed in two [47, 67] and EEG in one study [67].

#### Spine surgery

Surgical navigation systems were often used in the hybrid-OR (*n* = 18). These included augmented reality surgical navigation (ARSN) (ClarifEye Philips, The Netherlands; *n* = 10), cranial and spine navigation (Curve, Brainlab, Germany; *n* = 6, Stealth Station, Medtronic USA; *n* = 1), and the renaissance robotic navigation platform (Mazor Robotics Ltd., Caesarea, Israel, *n* = 1). Two studies combined robotic and ARSN [2, 7]. Two cadaveric studies compared the screw placement accuracy in ARSN-guided procedures with traditional methods [20, 60]. Results showed that the ARSN was non-inferior to fluoroscopy for the placement of pedicle screws [60], and reached a significantly higher accuracy than the free-hand technique [20]. In two human studies, surgical navigation was associated with an increased pedicle screw density (possibly resulting in better long-term outcomes) [17], and increased clinical accuracy compared to the free-hand technique [23]. Robotic-assisted surgery was implemented in the hybrid-OR, with the introduction of the corresponding technology as a supplement to the hybrid-OR equipment. In one study [2], a robotic arm was designed to assist pedicle screw placement guided by ARSN. By combining robotic and augmented reality techniques, high clinical (100%) and technical accuracy were achieved. Artificial intelligence was tested and validated for the automatic segmentation of vertebrae, pedicle identification, and planning of surgical path [6]. Another study using the renaissance robotic navigation platform (Mazor Robotics Ltd., Caesarea, Israel) to assist in pedicle screw placement reported a high level of accuracy [62].

#### Other uses of additional technologies

Two studies performing skull-base tumor surgery mentioned the use of Brainlab navigation (Brainlab, Munich, Germany) as an adjunct in the hybrid-OR. In addition, one study employed ARSN for guidance during cranial biopsy and EVD insertion procedures. One study used ultrasound during ventriculoarterial shunt placement.

### Radiation exposure

Radiation exposure was addressed in 16 studies, but since the data was derived from 14 unique datasets, only 14 studies were considered in this section. Of these 14 studies, ten mentioned at least one occupational radiation protection strategy including keeping a safe distance from the scattered radiation source (the patient) or stepping out of the OR during image acquisition (*n* = 6), using radiation protection shields (*n* = 5), or wearing lead aprons (*n* = 2).

Five studies, all in spine surgery, measured and reported the radiation exposure to staff and/or patients without performing any comparison, while the other nine studies presented comparison groups (Table 2). Among the five studies that had not performed comparisons, three studies reported the patient radiation exposure in terms of effective dose, with one study including the patient entrance surface dose. The other studies reported only so-called patient dose index expressed in terms of air kerma: the air kerma-area product (often referred to as the dose-area product (DAP)), reference (point) air kerma, and entrance surface air kerma.

**Table 2:** Findings of studies comparing radiation exposure associated with different imaging modalities, dose protocols, and type of procedures

Study ID	Study design	Procedure	Group 1 vs. group 2	Radiation measure (physical quantity)	Individuals exposed	Lowest radiation exposure	Relative difference	Significance	Radiation protection
Comparison of radiation associated with different imaging systems									
Kageyama 2017	Observational	Posterior lumbar interbody fusion	Hybrid-OR (*n* = 12) (artis Zeego) vs. conventional C-arm (*n* = 5) (Veradius unity, release 1–1, Philips, Netherlands)	Patient dose index (air kerma in Gy)	Patient	Mobile C-arm	78% reduction	***p* = 0.0052**	N/a
Cewe 2021	Observational	N/a	Hybrid-OR cone beam CT (hCBCT) (Allura Clarity) vs. mobile O-arm cone beam CT (oCBCT) system (Medtronic, Littleton, MA, USA	Occupational dose (personal dose equivalent, Hp(10) in Sv)	Staff	hCBCT	22% reduction on average, depending on the position in the hybrid-OR	***p* < 0.000**	Radiation protection shields
				Scattered dose (air kerma in Gy)		hCBCT	24% reduction on average, depending on the imaging protocol used	***p* < 0.05**	
Nachabe 2019	Observational	N/a	Fixed C-arm (hCBCT) (Allura Clarity) vs Mobile O-arm (mCBCT) (Medtronic, Littleton, MA)	Patient dose index (air kerma in Gy)	Patient	hCBCT	hCBCT was 30–17% of the mCBCT for the small phantom, 64–30% for the medium phantom, and 80–37% for the large phantom	N/a	N/a
Comparison of radiation exposure when using different protection strategies									
Kim 2019	Observational	Coil embolization for unruptured intracranial aneurysms	Standard protection vs. reinforced protection (protected the operator side by installing adjustable C-shaped wide lead screen WD304 (MAVIG GmbH) + a wall-type protection shield was located in front of the anesthesiologist in the direction of the radiation source)	Occupational dose (personal dose equivalent, Hp(10) in Sv)	Operator	Reinforced protection	41% reduction	*p* = NS	- Radioprotective vest-skirt aprons with a thyroid collar (all personnel) - Radioprotective cap and goggles (operator) - Lead shields - Radiation protection shield
					Anesthesiologist	Reinforced protection	85% reduction	*p* = NS	
					Nurse		68% reduction	*p* = NS	
					Radiologic technologist	Standard protection	62% reduction	*p* = NS	
				Occupational dose rate (personal dose equivalent rate, Hp(10) in Sv/s)	Operator	Reinforced protection	52% reduction	***p* = 0.04**	
					Anesthesiologist		55% reduction	*p* = NS	
					Nurse		78% reduction	*p* = **0.01**	
					Radiologic technologist		20%	*p* = NS	
Comparison of radiation associated with different dose protocols									
Pireau 2017	Randomized control trial	Pedicle screw placement	High dose (397 projection images during 6 s) vs. low dose (133 projection images during 5 s)	Patient dose index (air kerma in Gy)	Patient	Low dose	52% reduction	NM	N/a
				Patient skin dose (entrance surface dose in Gy)			80% reduction		
Edström 2019	Observational	Pedicle screw placement	Small field of view protocol (*n* = 10) (19 small and 19 medium field-of-view CBCT) vs. large field of view protocol (*n* = 10) (1 small, 21 medium, and 17 large field-of-view)	Patient dose index (air kerma-area product, KAP in Gy m^2^)	Patient	Large field of view protocol	52% reduction	***p* < 0.05**	N/a
				Patient dose (effective dose in mSv)			32% reduction	*p* = NS	
Kaminski 2017	Observational	Thoracic or lumbar fusion (pedicle screws or interbody spacers implantation)	High dose (397 projection images during 6 s) (*n* = 69) vs. low dose (133 projection images during 5 s) (*n* = 16)	Patient dose index (air kerma-area product, KAP, in Gy m^2^)	Patient	Low dose	78% reduction	***p* < 0.05**	N/a
				Patient dose index (air kerma in Gy)				***p* < 0.05**	
Comparison of radiation associated with different procedures									
Schuetze 2019	Observational	- Dorsal instrumentation of the spine (most common) - Sacroiliac screws - Combined pelvic and spinal procedures - Complex trauma procedures	Navigated procedures (*n* = 10) vs, non-navigated procedures (*n* = 4)	Occupational dose (personal dose equivalent, Hp(10) in Sv)	Surgeon	Navigated procedures	68% reduction	***p* = 0.002**	- Lead aprons - Collimation - Radiation protection shield - Steeping out of the OR
			Minimally invasive procedures (*n* = 34) vs. open procedures (*n* = 7)			Minimally invasive procedures	8% reduction	*p* = NS	
Kaminski 2017	Observational	Thoracic or lumbar fusion (pedicle screws or interbody spacers implantation)	Minimally invasive procedures (*n* = 38) vs. conventional procedures (*n* = 59)	Patient dose index (air kerma in Gy)	Patient	Conventional procedures	50% reduction	***p* < 0.05**	N/a
				Patient dose index (air kerma-area product, KAP, in Gy m^2^)					
			Minimally invasive procedures with navigation (*n* = 22) vs. minimally invasive procedures without navigation (*n* = 16)	Patient dose index (air kerma-area product, KAP, in Gy m^2^)		Without navigation	< 1% difference	*p* = NS	
			Conventional procedures with navigation (*n* = 16) vs. conventional procedures without navigation (*n* = 43)			Without navigation	22% reduction	*p* = NS	
Neki 2020	Observational	Dural arteriovenous fistulas repair	Direct trans-sinus embolization (dTSE) with craniotomy (*n* = 5) vs. transvenous embolization (TVE) (*n* = 6)	Patient dose index (air kerma in Gy)	Patient	dTSE	32% reduction	*p* = NS	N/a

*N/a* not applicable, *NS* non-significant

Three studies compared the radiation exposure from different intraoperative imaging systems. In one study, a ceiling-mounted robotic C-arm (AlluraClarity, Philips, The Netherlands) was compared with a mobile O-arm [50] (Medtronic, Littleton, MA, USA), indicating that the former was associated with a statistically significant reduction of 22% in scatter radiation relating to the occupational exposure [11]. Another study compared fluoroscopy alone (mobile C-arm, Veradius unity, Philips, The Netherlands) to fluoroscopy and CBCT (Artis Zeego, Siemens Healthcare, Germany) in a hybrid-OR and not surprisingly found a higher exposure in the latter case [32].

Three studies compared radiation exposure from different image acquisition protocols. As expected, the protocol with lower dose settings (133 projection images per CBCT rotation) significantly reduced radiation exposure when compared to a protocol with higher dose settings (397 projection images per CBCT rotation) during spine instrument implantation [33, 61]. In a third study, the use of large field of view protocol during pedicle screw placement was significantly associated with a 32% reduction in average effective dose as compared to the small field of view [16].

Two studies compared the radiation exposure to patient [33] and surgeon [64] during minimally invasive and open procedures of the spine. The first study found a statistically significant twofold decrease in dose-area product and air kerma in favor of conventional open procedures [33] while the second showed no significant differences regarding the surgeon’s exposure between the approaches [64]. Both studies also assessed the influence of navigation systems on radiation exposure. Navigation-assisted procedures were associated with a significantly reduced surgeon’s radiation dose [64] and a similar dose-area product [33] compared to non-navigated procedures.

### Advantages of the hybrid operating room

Sixty-nine studies (93%) discussed the advantages of the hybrid-OR (Table 3). The most consistently referred advantage (*n* = 24 studies) was the ability to immediately assess outcomes. Examples included confirmation of safe aneurysm clip positioning, complete obliteration of aneurysm [10, 12, 14, 28, 31, 49, 54, 66, 67, 72, 74] or fistulae [14, 29, 35, 48, 72, 77], and correct screw placement [4, 9, 17, 21, 25, 26, 32, 33, 52, 61, 69]. This could be associated with a decreased rate of revision surgery (*n* = 17) and a reduced need for routine postoperative imaging (*n* = 5).

**Table 3:** Advantages and disadvantages of hybrid operating rooms reported by studies

Advantage	Number of studies	Disadvantage	Number of studies
Immediate assessment of outcomes	24	Limited patient positioning	8
Ability to perform one stage combined endovascular and open treatment	19	Radiation exposure	7
Decreased rate of revision surgery	17	Prolonged procedural time	5
Increased procedural accuracy	12	Sterility issues	4
Reduced need for patient's movement	10	Necessity of proper training	4
Improved patient safety	10	High expenses	3
Reduced need for postoperative imaging	5	Requirement for a larger surgical space	2
Improved surgical results	4	–	–
Rapid detection of complications	4	–	–
Versatility of the hybrid-OR	3	–	–

The ability to perform combined endovascular and open procedures during a single session and in the same room was an important benefit of a hybrid-OR (*n* = 19). The possibility of seamless conversion to open surgical access in the event of failure or complication of the endovascular approach, without having to move the patient (*n* = 10), highlighted the role of hybrid-OR in improving patient safety (*n* = 10). In fact, rapid detection of complications (*n* = 4) was one of the advantages raised with the use of a hybrid-OR.

Other advantages of the hybrid-OR that were mentioned include, but are not limited to, improved surgical results (*n* = 4), increased accuracy of the procedure (*n* = 12), and versatility of the facility as hybrid-ORs can be utilized by a wide array of interventional and surgical disciplines (*n* = 3).

### Limitations of the hybrid operating room

In total, 37 studies discussed the limitations associated with hybrid-ORs (Table 3). The most frequently mentioned was the limited range of motion of the operating tables which may cause limitations for optimal patient positioning (*n* = 8). Similarly, one study reported the inability to adequately position obese patients in the hybrid-OR, mainly due to the limited gantry size of the C-arm [22]. Another consideration was radiation exposure and the necessity to properly monitor the radiation dose and to implement protection strategies (*n* = 7). Pitfalls in workflow are mostly immanent to the integration of cumbersome imaging systems into the operating room. Sterility issues (*n* = 4) and the requirement of a lager surgical space (*n* = 2) were both commonly mentioned.

Work in a hybrid-ORs requires proper training for the surgeons and the medical staff to attain optimal utilization of the equipment and to optimize the surgical workflow. Prolonged procedure time (*n* = 5) due to learning curve on the hybrid equipment is a common concern (*n* = 4). Although no exact costs were mentioned, higher expenses associated with the installation of a hybrid-OR has been reported (*n* = 3).

### Hybrid vs non-hybrid ORs

Merely three studies adopted a hybrid-vs-non-hybrid study design, where clinical or surgical success was the main outcome (Table 4). In one study [32], the use of a hybrid-OR equipped with a multi-axis angiography system increased pedicle screw accuracy, compared to the use of a mobile C-arm. A case–control study assessed the benefits of performing microsurgical clipping of ruptured aneurysms in the hybrid-OR with intraoperative angiography, over the conventional approach where only micro-doppler and ICGV fluorescence are used to control surgical outcomes [13]. The matched-pair analysis showed that treatment in the hybrid-OR implied an increased operative time with no significant improvement in clinical outcomes, such as aneurysm remnant, vessel occlusion, revision procedure, or mortality rates, individually. However, when regarded as a composite outcome, the results turned in favor of the hybrid-OR. In a technical report, the authors described their experience with a new fluoroscopy-guided technique for the placement of ventriculoperitoneal shunt in a hybrid-OR compared to the standard procedure relying on anatomical landmarks. Although the novel technique allowed accurate shunt placement, and a decrease of the associated early revision rate was speculated, no statistical analyses were provided [39].

**Table 4:** Findings of studies comparing hybrid and non-hybrid operating rooms

Study ID	Study design	Procedure	Group 1/experimental	Group 2/control	Important findings	Significance
Kobayashi 2012	Retrospective observational Case presentation	Ventriculoperitoneal shunt placement	New technique in hybrid-OR (*n* = 39)	Conventional method using external landmarks (*n* = 37)	Decreased revision and misplacement rate	N/a
Kageyama 2017	Retrospective observational	Posterior lumbar interbody fusion (PLiF) using percutaneous pedicle screws	Hybrid-OR (*n* = 12)	C-arm (*n* = 5) (conventional mobile C-arm Veradius unity)	Increased accuracy	***p* = 0.013**
					No difference in total operation time	*p* = NS
Dammann 2017	Case control	Microsurgical repair of ruptured intracranial aneurysm	Hybrid-OR (*n* = 20)	Conventional	Prolonged operation time	N/a
					Improved the combined outcome (scoring system based on radiological and functional outcomes)	***p* < 0.05**

*N/a* not applicable, *NS* non-significant

## Discussion

This systematic review finds that hybrid-ORs equipped with CBCT systems have been increasingly used in neurosurgery over the past decades, especially in the fields of spine and cerebrovascular surgery. At the same time, a shift towards minimally invasive surgery (MIS) has taken place in many surgical specialties, and neurosurgery is no exception. Characterized by smaller incisions and a greater preservation of tissues, MIS inevitably results in limited surgical visibility. This is generally compensated for using intraoperative imaging and navigation technologies. Whether the hybrid-OR provides the platform necessary to sustain the further development of MIS or if MIS will move towards mobile solutions remains to be seen. The publications analyzed in this review should not be taken to directly correspond to the everyday clinical use of the hybrid-OR. Rather, they reflect research interests relating to the technologies and use of the hybrid-OR. As such they may also provide an insight into possible future applications. In this context, it is notable that most of the publications stem from Europe and Asia and only a minority originates in the USA.

For cerebrovascular surgery, hybrid-ORs are equipped with high-end angiography systems comparable to those in a conventional angio-suite [28]. This provides the opportunity to perform imaging, including angiography, to confirm that the treatment goals have been met. Several studies have shown that intraoperative angiography is superior for the detection of remaining remnant vessel abnormalities compared to the alternatives: visual inspection, Doppler ultrasonography, and ICGV [27, 29, 43]. ICGV is commonly used at neurovascular centers as it is easy to use and does not expose the patient and staff to radiation. However, it has technical limitations. It relies on fluorescence necessitating a microscope equipped with the proper filters. Furthermore, the signal is poorly transmitted through tissues and consequently provides reliable information only regarding surface vasculature directly visualized in the microscope. In addition, as ICG is quickly distributed in the bloodstream but not rapidly washed away, it does not allow repeated boluses to be injected to effectively analyze how different interventions may affect blood flow.

One of the most important advantages of the hybrid-OR in the field of cerebrovascular surgery is the ability to perform a combined endovascular and open surgical approach without having to perform time-consuming transfers of the patient between the OR and angiography suite [37]. Combined approaches have gained popularity for the management of complex cerebrovascular disorders such as giant intracranial aneurysms and AVMs [49]. A recent study, published after the inclusion period, validated the feasibility and safety of combined procedures in a hybrid-OR for the treatment of pediatric cerebrovascular diseases [75]. Moreover, a hybrid environment allows for near-instantaneous detection and reaction to intraoperative complications—as conversion from endovascular to open procedure is a conceivable option in a hybrid-ORs. In fact, several studies reported performing rescue surgeries that would not have been possible in a conventional angiography suite [31, 71]. However, intraoperative angiography requires arterial access and is normally done with the patient supine which may be in conflict with the positioning needs of the surgical approach. Moreover, the periprocedural use of antithrombotic or anticoagulant drugs routinely used in neurointerventional procedures must be managed in relation to the neurosurgical risks.

In spine surgery, intraoperative imaging can reduce the risk of instrumentation-related neurovascular injury as well as screw misplacement [41]. Traditionally, intraoperative imaging in spinal procedures is performed using 2D fluoroscopy. However, navigation systems using intraoperatively acquired 3D images provide superior guidance with accurate, real-time tracking of the patient’s anatomy and surgical instruments. Intraoperative imaging and surgical navigation allow OR staff and surgeons to avoid radiation exposure and work without lead aprons but may increase the exposure to the patient [64]. Based on the studies included in this review, it can be concluded that navigated pedicle screw placements in a hybrid-OR is safe and more accurate than both fluoroscopy-assisted and free-hand techniques.

The combination of technologies in the hybrid-OR has been particularly studied in spine surgery. For instance, the ARSN system, specifically developed for the hybrid-OR, uses adhesive markers to create a virtual reference and provides an alternative to conventional tracking systems used for spinal navigation [8]. The incorporation of surgical robots [2, 62] may enhance surgical precision and patient safety and consequently increase the utility of the hybrid-OR [34, 63].

Unfortunately, only a few of the included studies investigated radiation exposure [36, 51]. The use of intraoperative CBCT for navigation or at end of procedure adds substantially to the patient’s radiation exposure. However, the patient dose must be calculated with consideration of the total dose related to the care provided [5]. A preoperative scan in the hybrid-OR provides better information for navigation than a preoperative one, and end-of procedure scan can replace a conventional postoperative scan [9]. To achieve the lowest patient doses, it is important that the hybrid-OR imaging equipment incorporates optimized x-ray imaging protocols and is used by trained staff. In a randomized control trial, a low-dose protocol was shown to significantly reduce the exposure without any compromise to the surgical accuracy [61].

Occupational exposure is reduced by avoiding unnecessary use of radiation, minimizing exposure time, maintaining maximal distance to the scatter source, and using radiation protection shielding [46]. This can be achieved through the implementation of local safety guidelines and proper training of the OR staff [32, 52]. Radioprotective shields, aprons, and garments can effectively reduce the exposure, but wearables are uncomfortable and may interfere with the surgeon’s work [1]. The possibility to remotely control the imaging systems, especially during CBCT acquisitions, enables the staff to protect themselves by either stepping out of the room or by standing behind protective lead shields. Navigated surgery lends itself well to these principles allowing staff exposure to be reduced to background radiation levels [25].

While hybrid-ORs provide an advantage in procedures where image guidance and navigation are necessary, the scientific literature also includes procedures without a specific need for this technology. Although no extensive cost–benefit analyses have been performed, cost concerns have been previously raised by some authors. Currently, however, hybrid-ORs are mostly found in academic centers and university hospitals, where the additional costs may be accepted in favor of research and technological advances as well as potential long-term benefits. Other concerns with respect to the use of hybrid-ORs that were poorly addressed within the covered literature mainly pertain to the training of staff and the associated learning curves.

### Future perspectives

Hybrid-ORs provide advanced intraoperative imaging capabilities as well as the possibility to combine endovascular and open surgical approaches for the management of complex neurosurgical cases. In this era of rapid technological advances, hybrid-ORs are likely to improve and evolve in terms of workflow, imaging technologies, and auxiliary technologies. This will provide neurosurgeons with the opportunity to treat more challenging cases and contribute to the advancement of minimally invasive, endovascular, and endoscopic approaches. For instance, it has been postulated that the adjunct of augmented reality and robotic techniques may enhance precision and improve patient safety during minimally invasive procedures [2, 45]. Similarly, endoscopic procedures may benefit from surgical guidance offered by the intraoperative imaging available in the hybrid OR [55].

Furthermore, the hybrid OR may provide an ideal environment for the multidisciplinary aspect of trauma management. Hybrid environments where neurosurgeons, vascular surgeons, and trauma surgeons collaborate with interventional radiologists manage patients with multiple injuries have already been described [38, 53, 56]. The increased availability of hybrid-ORs at major trauma centers may contribute to improved management and better patient outcomes by allowing immediate assessment of performed interventions, such as hematoma evacuations or spinal fixations. Additional measures could be undertaken immediately without the need for back-and-forth transportation between facilities. In the future, a module-based hybrid OR combining different technologies that may be used for different types of procedures could be envisioned. This type of ORs could contain CBCT, MRI, ultrasound, and different image-guided navigation solutions. The auxiliary technologies available might be active robotics, endoscopic devices, mixed-reality solutions, AI-guided decision tools, telemedical communication, and tutoring applications for consultation and teaching.

Nonetheless, more research is needed to develop protocols and optimize the workflows for different scenarios. It is also important that future work focuses on assessing the impact of hybrid ORs on patient outcomes from both a clinical and a socioeconomic perspective.

### Limitations

The main limitation of this review relates to the limitations of the literature itself. The lack of standardized outcome measures makes the results difficult to interpret and compare across studies. Metrics of radiation exposure and doses were rarely standardized, uniform, or consistent among studies, impeding direct quantitative and meta-analytic comparisons or pooling of the published data. Estimating the actual cost-effectiveness of a hybrid-OR could not be achieved, as this information was nearly non-existent. Other poorly addressed concerns regarding the use of hybrid-ORs mainly pertain to the training of staff and the associated learning curves. Due to the lack of objective metrics, addressing the effects of the learning curves associated with the introduction of hybrid-ORs was not possible. The learning curves depend on the complexity and degree of integration of new technologies in the hybrid-OR, on the previous training of the staff and the number of cases available for staff training, making this parameter difficult to evaluate. Albeit challenging, a thorough analysis of this aspect is important to justify the increasing use of hybrid-ORs. Furthermore, only a few studies compared hybrid-ORs to more conventional ORs. Randomized control trials to evaluate the efficacy and safety of hybrid-ORs are needed, as are studies aimed at determining which procedures and what patient categories benefit the most from treatment in hybrid-ORs. In fact, one study reached the conclusion that the hybrid-OR was only crucial to 2% of cerebrovascular procedures [42]. A study by Ogiwara et al., published after the inclusion period, compared CBCT-based hybrid-ORs to operating rooms equipped with other imaging devices and concluded that different procedures require surgical suites with different properties [55]. The authors reported that 12% of the neurosurgical procedures at their institution were conducted in a CBCT-based hybrid OR mostly concerning spine cases. Similar studies are needed to define the specifications of different hybrid-OR setups. This information would assist stakeholders in choosing what equipment better suits the specific needs of the hospital. Finally, funding in research on novel technologies may be provided by manufacturers, causing a potential source of bias. In this review, however, the risk of bias associated with each of the studies and its design was thoroughly assessed and provided as supplementary (Supplementary file 5).

## Conclusion

Hybrid-ORs, equipped with CBCT and angiography, are increasingly used in the context of both vascular and spinal neurosurgery. They provide preprocedure, intraprocedure, and end-of-procedure imaging capabilities, thereby increasing the surgical precision, and reducing the need for postoperative imaging and correction surgeries. Despite these advantages, prolonged operative durations and radiation exposure to patient and staff are important concerns. However, protective measures may result in reduced exposure for both patient and staff compared to conventional solutions. Hybrid-ORs offer possibilities in the development of new minimally invasive surgical approaches, but the costs of installation and equipment acquisition limit their current use to large centers. Given the lack of randomized, controlled studies objectively evaluating the superiority of hybrid-ORs, the current state of the literature indicates that the use of hybrid ORs in neurosurgery is still in an experimental and developmental phase.

## Supplementary Information

Below is the link to the electronic supplementary material.

Supplementary file1 (DOCX 128 KB)

Supplementary file2 (PDF 128 KB)

Supplementary file3 (DOCX 79 KB)

Supplementary file4 (DOCX 453 KB)

Supplementary file5 (DOCX 111 KB)

## References

[1] Alexandre D, Prieto M, Beaumont F, Taiar R, Polidori G (2017). Wearing lead aprons in surgical operating rooms: ergonomic injuries evidenced by infrared thermography. J Surg Res.

[2] Balicki M, Kyne S, Toporek G (2020). Design and control of an image-guided robot for spine surgery in a hybrid OR. Int J Med Rob Comput Assisted Surg.

[3] Barstad RM, Fosse E, Vatne K, Andersen K, Tønnessen TI, Svennevig JL, Geiran OR (1997). Intraoperative angiography in minimally invasive direct coronary artery bypass grafting. Ann Thorac Surg.

[4] Bohoun CA, Naito K, Yamagata T, Tamrakar S, Ohata K, Takami T (2019). Safety and accuracy of spinal instrumentation surgery in a hybrid operating room with an intraoperative cone-beam computed tomography. Neurosurg Rev.

[5] Brambilla M, Vassileva J, Kuchcinska A, Rehani MM (2020). Multinational data on cumulative radiation exposure of patients from recurrent radiological procedures: call for action. Eur Radiol.

[6] Burström G, Buerger C, Hoppenbrouwers J (2019). Machine learning for automated 3-dimensional segmentation of the spine and suggested placement of pedicle screws based on intraoperative cone-beam computer tomography. J Neurosurg Spine.

[7] Burström G, Balicki M, Patriciu A (2020). Feasibility and accuracy of a robotic guidance system for navigated spine surgery in a hybrid operating room: a cadaver study. Sci Rep.

[8] Burström G, Nachabe R, Homan R, Hoppenbrouwers J, Holthuizen R, Persson O, Edström E, Elmi-Terander A (2020). Frameless patient tracking with adhesive optical skin markers for augmented reality surgical navigation in spine surgery. Spine (Phila Pa 1976).

[9] Burström G, Cewe P, Charalampidis A, Nachabe R, Söderman M, Gerdhem P, Elmi-Terander A, Edström E (2021). Intraoperative cone beam computed tomography is as reliable as conventional computed tomography for identification of pedicle screw breach in thoracolumbar spine surgery. Eur Radiol.

[10] Byval’tsev VA, Belykh EG, Kikuta KI, Stepanov IA (2018). A hybrid neurosurgical operating room: potentials in the treatment of arteriovenous malformations of the brain. Biomed Eng (NY).

[11] Cewe P, Vorbau R, Omar A, Elmi-Terander A, Edström E (2021) Radiation distribution in a hybrid operating room, utilizing different X-ray imaging systems: investigations to minimize occupational exposure. J Neurointerv Surg Neurintsurg-2021–018220 10.1136/neurintsurg-2021-018220 PMC9606514 34750111

[12] Choi E, Lee JY, Jeon HJ, Cho BM, Yoon DY (2019). A hybrid operating room for combined surgical and endovascular procedures for cerebrovascular diseases: a clinical experience at a single centre. Br J Neurosurg.

[13] Dammann P, Jägersberg M, Kulcsar Z, Radovanovic I, Schaller K, Bijlenga P (2017). Clipping of ruptured intracranial aneurysms in a hybrid room environment—a case-control study. Acta Neurochir (Wien).

[14] Durner G, Wahler H, Braun M, Kapapa T, Wirtz CR, König R, Pala A (2021). The value of intraoperative angiography in the time of indocyanine green videoangiography in the treatment of cerebrovascular lesions: efficacy, workflow, risk-benefit and cost analysis A prospective study. Clin Neurol Neurosurg.

[15] Edström E, Burström G, Nachabe R, Gerdhem P, Terander AE (2020). A novel augmented-reality-based surgical navigation system for spine surgery in a hybrid operating room: design, workflow, and clinical applications. Operative Surg.

[16] Edström E, Burström G, Omar A, Nachabe R, Söderman M, Persson O, Gerdhem P, Elmi-Terander A (2020). Augmented reality surgical navigation in spine surgery to minimize staff radiation exposure. Spine (Phila Pa 1976).

[17] Edström E, Burström G, Persson O, Charalampidis A, Nachabe R, Gerdhem P, Elmi-Terander A (2020). Does augmented reality navigation increase pedicle screw density compared to free-hand technique in deformity surgery? Single Surgeon Case Series of 44 Patients. Spine (Phila Pa 1976).

[18] El-Hajj VG, Pettersson-Segerlind J, Fletcher-Sandersjöö A, Edström E, Elmi-Terander A (2022). Current knowledge on spinal meningiomas epidemiology, tumor characteristics and non-surgical treatment options: a systematic review and pooled analysis (part 1). Cancers (Basel).

[19] El-Hajj VG, Pettersson-Segerlind J, Fletcher-Sandersjöö A, Edström E, Elmi-Terander A (2022). Current knowledge on spinal meningiomas—surgical treatment, complications, and outcomes: a systematic review and meta-analysis (Part 2). Cancers (Basel).

[20] Elmi-Terander A, Skulason H, Soderman M, Racadio J, Homan R, Babic D, van der Vaart N, Nachabe R (2016). Surgical navigation technology based on augmented reality and integrated 3D intraoperative imaging a spine cadaveric feasibility and accuracy study. Spine (Phila Pa 1976).

[21] Elmi-Terander A, Nachabe R, Skulason H, Pedersen K, Söderman M, Racadio J, Babic D, Gerdhem P, Edström E (2018). Feasibility and accuracy of thoracolumbar minimally invasive pedicle screw placement with augmented reality navigation technology. Spine (Phila Pa 1976).

[22] Elmi-Terander A, Burström G, Nachabe R (2019). Pedicle screw placement using augmented reality surgical navigation with intraoperative 3D imaging: a first in-human prospective cohort study. Spine (Phila Pa 1976).

[23] Elmi-Terander A, Burström G, Nachabé R, Fagerlund M, Ståhl F, Charalampidis A, Edström E, Gerdhem P (2020). Augmented reality navigation with intraoperative 3D imaging vs fluoroscopy-assisted free-hand surgery for spine fixation surgery: a matched-control study comparing accuracy. Sci Rep.

[24] Fierstra J, Anon J, Mendelowitsch I, Fandino J, Diepers M, Remonda L, Marbacher S (2020). Amended intraoperative neuronavigation: three-dimensional vascular roadmapping with selective rotational digital subtraction angiography. World Neurosurg.

[25] Fomekong E, Safi SE, Raftopoulos C (2017). Spine navigation based on 3-dimensional robotic fluoroscopy for accurate percutaneous pedicle screw placement: a prospective study of 66 consecutive cases. World Neurosurg.

[26] Fong YW, Su IC, Hsieh CT, Huang CT, Chang CJ (2020). Accuracy and safety of pedicle screws implantation using Zeego and Brainlab navigation system in hybrid operation room. Formos J Surg.

[27] Goren O, Bourdages G, Schirmer CM, Weiner G, Dalal SS, Griessenauer CJ (2020). Intraoperative 3-dimensional rotational angiography in cerebrovascular surgery: a case series. World Neurosurg.

[28] Grüter BE, Mendelowitsch I, Diepers M, Remonda L, Fandino J, Marbacher S (2018). Combined endovascular and microsurgical treatment of arteriovenous malformations in the hybrid operating room. World Neurosurg.

[29] Grüter BE, Strange F, Burn F, Remonda L, Diepers M, Fandino J, Marbacher S (2018). Hybrid operating room settings for treatment of complex dural arteriovenous fistulas. World Neurosurg.

[30] Iop A, El-Hajj VG, Gharios M, de Giorgio A, Monetti FM, Edström E, Elmi-Terander A, Romero M (2022). Extended reality in neurosurgical education: a systematic review. Sensors.

[31] Jeon HJ, Lee JY, Cho BM, Yoon DY, Oh SM (2019). Four-year experience using an advanced interdisciplinary hybrid operating room : potentials in treatment of cerebrovascular disease. J Korean Neurosurg Soc.

[32] Kageyama H, Yoshimura S, Uchida K, Iida T (2017). Advantages and disadvantages of multi-axis intraoperative angiography unit for percutaneous pedicle screw placement in the lumbar spine. Neurol Med Chir (Tokyo).

[33] Kaminski L, Cordemans V, Cartiaux O, van Cauter M (2017). Radiation exposure to the patients in thoracic and lumbar spine fusion using a new intraoperative cone-beam computed tomography imaging technique: a preliminary study. Eur Spine J.

[34] Kantelhardt SR, Martinez R, Baerwinkel S, Burger R, Giese A, Rohde V (2011). Perioperative course and accuracy of screw positioning in conventional, open robotic-guided and percutaneous robotic-guided, pedicle screw placement. Eur Spine J.

[35] Kato N, Ishibashi T, Maruyama F (2021). Clinical outcomes of procedures combining endovascular embolization with a direct surgical approach in a hybrid operating room for the treatment of refractory dural arteriovenous fistulas. Surg Neurol Int.

[36] Kim T, Kwon OK, Ban SP, Kim YD, Won YD (2019). A phantom menace to medical personnel during endovascular treatment of cerebral aneurysms: real-time measurement of radiation exposure during procedures. World Neurosurg.

[37] Kim CH, Lee SW, Kim YH, Sung SK, Son DW, Song GS (2020). The experience of surgery and endovascular procedure of cerebrovascular disease in the hybrid operating room; multi-axis robotic C-arm DSA system. J Cerebrovasc Endovasc Neurosurg.

[38] Kinoshita T, Hayashi M, Yamakawa K, Watanabe A, Yoshimura J, Hamasaki T, Fujimi S (2018). Effect of the hybrid emergency room system on functional outcome in patients with severe traumatic brain injury. World Neurosurg.

[39] Kobayashi S, Ishikawa T, Mutoh T, Hikichi K, Suzuki A (2012). A novel technique for ventriculoperitoneal shunting by flat panel detector CT-guided real-time fluoroscopy. Surg Neurol Int.

[40] Kumar M, Shanavas M, Sidappa A, Kiran M (2015). Cone beam computed tomography—know its secrets. J Int Oral Health.

[41] Kumar V, Baburaj V, Patel S, Sharma S, Vaishya R (2021). Does the use of intraoperative CT scan improve outcomes in Orthopaedic surgery? A systematic review and meta-analysis of 871 cases. J Clin Orthop Trauma.

[42] Liao CH, Chen WH, Lee CH, Shen SC, Tsuei YS (2019). Treating cerebrovascular diseases in hybrid operating room equipped with a robotic angiographic fluoroscopy system: level of necessity and 5-year experiences. Acta Neurochir (Wien).

[43] Marbacher S, Steiger HJ (2021). Letter: commentary: value of 3-dimensional digital subtraction angiography for detection and classification of intracranial aneurysm remnants after clipping. Oper Neurosurg.

[44] Marbacher S, Kienzler JC, Mendelowitsch I, D’Alonzo D, Andereggen L, Diepers M, Remonda L, Fandino J (2020). Comparison of intra- and postoperative 3-dimensional digital subtraction angiography in evaluation of the surgical result after intracranial aneurysm treatment. Neurosurgery.

[45] McClendon J, Almekkawi AK, Abi-Aad KR, Maiti T (2020). Use of pheno room, augmented reality, and 3-rod technique for 3-dimensional correction of adolescent idiopathic scoliosis. World Neurosurg.

[46] Mountford PJ, Temperton DH (1992). Recommendations of the International Commission on Radiological Protection (ICRP) 1990. Eur J Nucl Med.

[47] Murayama Y, Saguchi T, Ishibashi T, Ebara M, Takao H, Irie K, Ikeuchi S, Onoue H, Ogawa T, Abe T (2006). Endovascular operating suite: future directions for treating neurovascular disease. J Neurosurg.

[48] Murayama Y, Irie K, Saguchi T (2011). Robotic digital subtraction angiography systems within the hybrid operating room. Neurosurgery.

[49] Murayama Y, Arakawa H, Ishibashi T (2013). Combined surgical and endovascular treatment of complex cerebrovascular diseases in the hybrid operating room. J Neurointerv Surg.

[50] Nachabe R, Strauss K, Schueler B, Bydon M (2019). Radiation dose and image quality comparison during spine surgery with two different, intraoperative 3D imaging navigation systems. J Appl Clin Med Phys.

[51] Neki H, Yonezawa A, Shibata A, Tsukagoshi E, Yamane F, Ishihara S, Kohyama S (2020). A minimally invasive approach for the treatment of isolated type intracranial dural arteriovenous fistula in a neurosurgical hybrid operating room. Interdiscip Neurosurg.

[52] Nevzati E, Fandino J, Schatlo B, Heimberg M, Marbacher S, Remonda L, Fathi AR (2017). Validation and accuracy of intraoperative CT scan using the Philips AlluraXper FD20 angiography suite for assessment of spinal instrumentation. Br J Neurosurg.

[53] Nguyen HS, Oni-Orisan A, Doan N, Mueller W (2016). Transnasal penetration of a ballpoint pen: case report and review of literature. World Neurosurg.

[54] Nossek E, Chalif DJ, Buciuc R, Gandras EJ, Anderer EG, Insigna S, Dehdashti AR, Setton A (2017). Intraoperative angiography for arteriovenous malformation resection in the prone and lateral positions, using upper extremity arterial access. Oper Neurosurg.

[55] Ogiwara T, Miyaoka Y, Nakamura T, Tsukada K, Yamazaki D, Ito K, Hanaoka Y, Koyama JI, Horiuchi T, Hongo K (2019). Endoscopic endonasal odontoidectomy in the hybrid operating room. World Neurosurg.

[56] Okada K, Tanei T, Kato T, Naito T, Koketsu Y, Ito R, Hirayama K, Hasegawa T (2022). Achieving good neurological outcome by combining decompressive craniectomy for acute subdural hematoma and transarterial embolization of intraperitoneal injured arteries for multiple severe trauma: a case report. Nagoya J Med Sci.

[57] Ouzzani M, Hammady H, Fedorowicz Z, Elmagarmid A (2016). Rayyan-a web and mobile app for systematic reviews. Syst Rev.

[58] Page MJ, McKenzie JE, Bossuyt PM (2021). The PRISMA 2020 statement: an updated guideline for reporting systematic reviews. BMJ.

[59] Pauwels R, Araki K, Siewerdsen JH, Thongvigitmanee SS (2015). Technical aspects of dental CBCT: state of the art. Dentomaxillofac Radiol.

[60] Peh S, Chatterjea A, Pfarr J, Schäfer JP, Weuster M, Klüter T, Seekamp A, Lippross S (2020). Accuracy of augmented reality surgical navigation for minimally invasive pedicle screw insertion in the thoracic and lumbar spine with a new tracking device. Spine J.

[61] Pireau N, Cordemans V, Banse X, Irda N, Lichtherte S, Kaminski L (2017). Radiation dose reduction in thoracic and lumbar spine instrumentation using navigation based on an intraoperative cone beam CT imaging system: a prospective randomized clinical trial. Eur Spine J.

[62] Schroeder JE, Houri S, Weil YA, Liebergall M, Moshioff R, Kaplan L (2022). When giants talk; robotic dialog during thoracolumbar and sacral surgery. BMC Surg.

[63] Schroerlucke SR, Wang MY, Cannestra AF, Good CR, Lim J, Hsu VW, Zahrawi F (2017). Complication rate in robotic-guided vs fluoro-guided minimally invasive spinal fusion surgery: report from MIS Refresh prospective comparative study. Spine J.

[64] Schuetze K, Eickhoff A, Dehner C, Schultheiss M, Gebhard F, Richter PH (2019). Radiation exposure for the surgical team in a hybrid-operating room. J Robot Surg.

[65] Shea BJ, Reeves BC, Wells G (2017). AMSTAR 2: a critical appraisal tool for systematic reviews that include randomised or non-randomised studies of healthcare interventions, or both. BMJ.

[66] Shimada K, Yamaguchi T, Miyamoto T, Sogabe S, Korai M, Okazaki T, Kanematsu Y, Satomi J, Nagahiro S, Takagi Y (2021). Efficacy of intraarterial superselective indocyanine green videoangiography in cerebral arteriovenous malformation surgery in a hybrid operating room. J Neurosurg.

[67] Song J, Li P, Tian Y (2021). One-stage treatment in a hybrid operation room to cure brain arteriovenous malformation: a single-center experience. World Neurosurg.

[68] Spenkelink IM, Heidkamp J, Fütterer JJ, Rovers MM (2022). Image-guided procedures in the hybrid operating room: a systematic scoping review. PLoS One.

[69] Tanikawa Y, Oba H, Fujii M (2022). Intraoperative cone beam CT in hybrid operation room for pediatric scoliosis patients. Spine (Phila Pa 1976).

[70] Tatter C, El-Hajj VG, Fletcher-Sandersjöö A, Edström E, Elmi-Terander A (2023). Radiographic measurements for the prediction of dysphagia after occipitocervical fusion: a systematic review. Acta Neurochir (Wien).

[71] Tsuei YS, Liao CH, Lee CH, Liang YJ, Chen WH, Yang SF (2018). Intraprocedural arterial perforation during neuroendovascular therapy: preliminary result of a dual-trained endovascular neurosurgeon in the neurosurgical hybrid operating room. J Chin Med Assoc.

[72] Wang C, Hsu SK, Chang CJ, Chen MH, Huang CT, Huang JS, Su IC (2020). Transfemoral approach for intraoperative angiography in the prone or three-quarter prone position: a revisited protocol for intracranial arteriovenous malformation and fistula surgery. Clin Neuroradiol.

[73] Wells GA, SheaD B, O’Connell D, Peterson J, Welch V, Losos M, Tugwell P (2000) The Newcastle-Ottawa Scale (NOS) for assessing the quality of nonrandomized studies in meta-analyses. https://www.ohri.ca/programs/clinical_epidemiology/oxford.asp. Accessed 24 Jul 2022

[74] Yu JL, Guo YB, Xu BF, Chen X, Xu K (2016). Onyx embolization and surgical removal as a treatment for hemorrhagic AVM in a hybrid operating room. Int J Clin Exp Med.

[75] Yue JK, Chang D, Caton MT (2022). The hybrid operative suite with intraoperative biplane rotational angiography in pediatric cerebrovascular neurosurgery: utility and lessons learned. Pediatr Neurosurg.

[76] Zakhary WZA, Ender JK (2018) Procedures in the hybrid operating room. Kaplan’s Essentials of Cardiac Anesthesia for Cardiac Surgery 534–550

[77] Zhang N, Xin WQ (2020). Application of hybrid operating rooms for treating spinal dural arteriovenous fistula. World J Clin Cases.

