# Supplementary material for: The use of hybrid operating rooms in neurosurgery, advantages, disadvantages, and future perspectives: a systematic review
Source: Acta Neurochir (Wien). 2023 Aug 16;165(9):2343–58. doi: 10.1007/s00701-023-05756-7 (PMC10477240; doi:10.1007/s00701-023-05756-7)
Supplement: Supplementary file 3 — Supplementary file3 (DOCX 79 KB) [file 701_2023_5756_MOESM3_ESM.docx]

Search strategies opted on each of the selected electronic databases

| **Database** | **Queries** |
| --- | --- |
| **PubMed** | (hybrid OR CBCT OR cone-beam) AND (operat* room) AND (neuro* OR cran* OR spin* OR skull) AND (surg* OR interven*) |
| **Embase** | (hybrid OR cbct OR 'cone beam*') AND 'operation room'/exp AND (neuro* OR cran* OR spin* OR skull) AND (surg* OR interven*) AND [english]/lim NOT ('proceeding' OR 'conference paper' OR 'conference abstract' OR 'review' OR 'case report') |
| **Web of Science** | (ALL=(Hybrid) OR ALL=(CBCT) OR ALL=(cone-beam*)) AND ALL=(operat* room) AND (ALL=(neuro*) OR ALL=(cran*) OR ALL=(spin*) OR ALL=(skull)) AND (ALL=(surg*) OR ALL=(interven*)) and English(Languages) and 18TH INTERNATIONAL CONGRESS OF THE INTERNATIONAL SOCIETY OF CRANIOFACIAL SURGERY ISCFS or 32ND ANNUAL MEETING OF THE EUROPEAN ASSOCIATION FOR CARDIO THORACIC SURGERY or 34TH ANNUAL MEETING OF THE NORTH EASTERN SOCIETY OF PLASTIC SURGEONS NESPS or SURGICAL MOTION PICTURE SESSION OF THE 45TH ANNUAL MEETING OF THE SOCIETY OF THORACIC SURGEONS (Exclude – Conference Titles) and Review Articles (Exclude – Document Types) |
