# Supplementary material for: The use of hybrid operating rooms in neurosurgery, advantages, disadvantages, and future perspectives: a systematic review
Source: Acta Neurochir (Wien). 2023 Aug 16;165(9):2343–58. doi: 10.1007/s00701-023-05756-7 (PMC10477240; doi:10.1007/s00701-023-05756-7)
Supplement: Supplementary file 4 — Supplementary file4 (DOCX 453 KB) [file 701_2023_5756_MOESM4_ESM.docx]

Table A. Baseline characteristics of studies included

| **Study ID** | **Location** | **Sample size** | **Type of surgery** | **Procedure(s)** | **Imaging system** | **CBCT use (s)** | **Additional technologies** |
| --- | --- | --- | --- | --- | --- | --- | --- |
| Kim 2020^1^ | Korea | 1832 sessions | - Cerebrovascular - Other | - Aneurysm treatment  - Carotid artery stenting  - Combined procedure for bow Hunter syndrome - Rescue surgery after endovascular procedures - Different sorts of shunt placement procedures - Intracerebral hemorrhage aspiration | Artis Zeego | - Interventional guidance - Intraoperative imaging and confirmation - Postoperative control | - |
| Ashour 2016^2^ | USA | 6 patients | - Cerebrovascular - Other | - Aneurysm treatment  - AVF treatment  - AVM treatment  - Intracerebral hemorrhage aspiration - EVD placement | Artis Zeego | - Preoperative imaging and surgical planning - Fluoroscopic guidance - Navigation - Postoperative control | - |
| Murayama 2013^3^ | Japan | 29 patients | - Cerebrovascular - Spinal | - Aneurysm treatment  - AVM treatment - AVF treatment  - Other non-neurosurgical procedures | - Axiom Artis dBA - Zeego | - Preoperative imaging - Fluoroscopic guidance - Postoperative control | - |
| Murayama 2010^4^ | Japan | 3 patients | - Cerebrovascular  - Spinal | - Spine instrumentation - AVM treatment  - Aneurysm treatment | Artis Zeego | - Preoperative imaging and surgical planning - Fluoroscopic guidance - Navigation  - Postoperative control | Syngo iGuide (Siemens Healthcasre, Forchheim, Germany |
| Schaller 2011^5^ | Switzerland | 728 procedures | - Cerebrovascular  - Spinal  - Skull base tumor surgery | - Aneurysm treatment - AVM treatment - AVF treatment - EC-IC bypass procedure - Skull-base tumor resection - Complex spine procedures | Allura FD20 | - Interventional guidance - Intraoperative imaging  - Navigation  - Postoperative control | Brainlab navigation system (Brainlab, Munich, Germany) |
| Irie 2008^6^ | Japan | 93 patients | - Spinal  - Cerebrovascular - Brain tumor surgery - Other | - Management of subarachnoid hemorrhage - AVM treatment  - Tumor resection - Carotid stenting  - Aneurysm treatment  - Angioma treatment  - AVF treatment  - Brain abscesses treatment  - Shunt placement - Unspecified spinal procedures - Other non-neurosurgical procedures | Artis BA and dBA | - Preoperative imaging and surgical planning - Fluoroscopic guidance  - Intraoperative imaging | - |
| Liao 2019^7^ | Taiwan | 1027 patients | Cerebrovascular | - Diagnostic angiography without surgery - Carotid artery stenting  - Aneurysm treatment  - AVM treatment  - Intracerebral hemorrhage aspiration - Thrombolysis/thrombectomy for ischemic stroke - Rescue surgery during embolization | Artis Zeego | - Preoperative imaging and surgical planning - Fluoroscopic guidance - Postoperative control | - |
| Dammann 2017^8^ | Switzerland | 40 patients | Cerebrovascular | Aneurysm treatment | Allura FD20 | - Postoperative control | - Micro-Doppler sonography - Indocyanine Green Video Angiography |
| Song 2021^9^ | China | 54 patients | Cerebrovascular | AVM treatment | Discovery IGS 730 (GE Healthcare) | - Interventional guidance  - Intraoperative imaging and confirmation  - Navigation | - Functional MRI - Ultrasound  - EEG |
| Choi 2022^10^ | South Korea | 6 patients | Cerebrovascular | - Aneurysm treatment  - Vertebral artery stenosis treatment | Unclear | - Fluoroscopic guidance | - |
| Yu 2016^11^ | China | 8 patients | Cerebrovascular | AVM treatment | Unclear | - Preoperative imaging and surgical planning - Interventional guidance - Intraoperative imaging - Postoperative control | - |
| Byval 2018^12^ | Russia | 1 patient | Cerebrovascular | AVM treatment | Allura Xper FD20 | - Preoperative imaging - Intraoperative imaging and confirmation - Navigation  - Postoperative control | - Indocyanine Green Video Angiography - Brainlab navigation system (Brainlab, Munich, Germany |
| Kienzler 2020^13^ | Switzerland | 3 patients | Cerebrovascular | Aneurysm treatment | Allura Xper FD20 | - Preoperative imaging  - Fluoroscopic guidance  - Postoperative control | - |
| Fierstra 2020^14^ | Switzerland | 1 patient | Cerebrovascular | Aneurysm treatment | Allura Xper FD20 | - Preoperative imaging and surgical planning - Intraoperative imaging to update the neuronavigation system - Navigation | - Brainlab navigation system (Brainlab, Munich, Germany - AR |
| Iihara 2013^15^ | Japan | 11 patients | Cerebrovascular | - Aneurysm treatment - Carotid endarterectomy and stenting | Allura Xper FD20 | - Fluoroscopic guidance - Postoperative control | Indocyanine Green Video Angiography |
| Zhang 2020^16^ | China | 11 patients | Cerebrovascular | AVF treatment | Unclear | - Preoperative imaging and surgical planning - Intraoperative imaging  - Postoperative control | - |
| Neki 2020^17^ | Japan | 11 patients | Cerebrovascular | AVF treatment | AXIOM Artis dBA | - Surgical planning - Interventional guidance | - |
| Zhang 2020^18^ | China | 12 patients | Cerebrovascular | Aneurysm treatment | Unclear | - Intraoperative imaging and confirmation - Interventional guidance - Postoperative control | - |
| Marbacher 2019^19^ | Switzerland | 120 patients | Cerebrovascular | Aneurysm treatment | Allura Xper FD20 | - Postoperative control | - Doppler sonography - Indocyanine Green Video Angiography |
| Kato 2021^20^ | Japan | 13 patients | Cerebrovascular | AVF treatment | Artis | - Preoperative imaging  - Fluoroscopic guidance  - Postoperative control | Indocyanine Green Video Angiography |
| Shimada 2020^21^ | Japan | 13 patients | Cerebrovascular | AVM treatment | Unclear | - Intraoperative imaging  - Postoperative control | Indocyanine Green Video Angiography |
| Kim 2019^22^ | Korea | 19 procedures | Cerebrovascular | Aneurysm treatment | INFX-8000V (Canon) | Not mentioned | - |
| Choi 2019^23^ | Korea | 191 procedures | Cerebrovascular | - Aneurysm treatment - AVM treatment - AVF treatment - Ischemic stroke surgical management - Intracerebral hemorrhage aspiration | Allura Xper FD20 | - Preoperative imaging  - Intraoperative imaging and confirmation - Navigation - Postoperative control | - Indocyanine Green Video Angiography - XperGuide System (Philips Medical Systems, Best, the Netherlands) |
| Marbacher 2021^24^ | Switzerland | 192 patients | Cerebrovascular | Aneurysm treatment | AlluraXper FD20 | - Postoperative control | - Doppler sonography - Indocyanine Green Video Angiography |
| Kawamura 2017^25^ | Japan | 2 patients | Cerebrovascular | Carotid endarterectomy | AlluraXper FD20 | - Interventional guidance  - Intraoperative imaging | Indocyanine Green Video Angiography |
| Xin 2021^26^ | China | 20 patients | Cerebrovascular | AVM treatment | UNIQ FD2020 (Philips) | - Interventional guidance - Postoperative control | - |
| Fong 2018^27^ | Taiwan | 24 patients | Cerebrovascular | Aneurysm treatment | Artis Zeego | - Preoperative imaging - Postoperative control | Indocyanine Green Video Angiography |
| Zheng 2013^28^ | China | 25 patients | Cerebrovascular | Aneurysm treatment | Toshiba | - Preoperative imaging and surgical planning - Interventional guidance  - Postoperative control | - |
| Marbacher 2020^29^ | Switzerland | 26 patients | Cerebrovascular | Aneurysm treatment | AlluraXper FD20 | - Postoperative control | - Doppler sonography - Indocyanine Green Video Angiography |
| Murayama 2006^30^ | Japan | 332 patients | Cerebrovascular | - Aneurysm treatment  - AVM treatment  - AVF treatment  - Tumor embolization  - Presurgical cerebrospinal angiography with or without subsequent surgical procedures | Axiom Artis BA and dBA | - Preoperative imaging  - Fluoroscopic guidance  - Intraoperative imaging  - Postoperative control | MRI |
| Goren 2020^31^ | Austria | 39 patients | Cerebrovascular | - Aneurysm treatment  - AVM treatment  - Bypass procedures  - AVF treatment | Unclear | - Postoperative control | - Doppler sonography - Indocyanine Green Video Angiography |
| Jiang 2019^32^ | China | 42 patients | Cerebrovascular | Carotid endarterectomy | Artis Zeego | - Interventional guidance - Intraoperative imaging  - Postoperative control | - |
| Jeon 2019^33^ | Korea | 49 procedures | Cerebrovascular | - Aneurysm treatment - AVM treatment - Intracerebral hemorrhage aspiration | Allura Xper 20FD | - Preoperative imaging - Intraoperative imaging - Navigation - Postoperative control | - Indocyanine green video angiography - XperGuide System (Philips Medical Systems, Best, the Netherlands) |
| Grüter 2018^34^ | Switzerland | 49 patients | Cerebrovascular | AVM treatment | Allura Xper FD2 | - Interventional guidance  - Intraoperative imaging - Navigation - Postoperative control | - Indocyanine green video angiography - Brainlab navigation system (Brainlab, Munich, Germany) |
| Grüter 2018^35^ | Switzerland | 8 patients | Cerebrovascular | AVF treatment | Allura Xper FD20 | - Interventional guidance  - Navigation - Postoperative control | - Doppler sonography  - Indocyanine green video angiography - Brainlab navigation system (Brainlab, Munich, Germany) |
| Nossek 2017^36^ | USA | 5 patients | Cerebrovascular | AVM treatment | Artis Zeego | - Fluoroscopic guidance  - Intraoperative imaging - Postoperative control | Ultrasound |
| Tsuei 2018^37^ | Taiwan | 5 patients | Cerebrovascular | Rescue procedures following intraprocedural arterial perforation | Artis Zeego | - Interventional guidance  - Intraoperative imaging  - Postoperative control | - |
| Park 2020^38^ | Korea | 50 patients | Cerebrovascular | Aneurysm treatment | Allura FD20 | - Postoperative control | - Doppler sonography - Indocyanine green video angiography |
| Durner 2021^39^ | Germany | 54 patients | Cerebrovascular | - Aneurysm treatment  - AVM treatment | Artis Zeego | - Intraoperative imaging and confirmation - Postoperative control | Indocyanine green video angiography |
| Wang 2020^40^ | China | 9 patients | Cerebrovascular | AVM treatment  AVF treatment | Artis Zeego | - Interventional guidance  - Intraoperative imaging  - Postoperative control | - |
| Fandino 2013^41^ | Switzerland | 99 patients | Cerebrovascular | - Aneurysm treatment  - AVM treatment | Allura Xper FD20 | - Interventional guidance  - Postoperative control | - |
| Skyrman 2021^42^ | Sweden | 40 procedures | Other | - Cranial biopsy - EVD insertion | Allura FlexMove | - Preoperative imaging and surgical planning  - Navigation - Postoperative control | ARSN |
| Kim 2020^43^ | Korea | 5 patients | Other | Ventriculo-atrial shunt placement | Artis Zeego | - Fluoroscopic guidance - Postoperative control | Ultrasound |
| Kobayashi 2012^44^ | Japan | 78 patients | Other | Ventriculoperitoneal shunt placement | AlluraXper FD20 | - Preoperative imaging and surgical planning  - Fluoroscopic guidance - Postoperative control | - |
| Mori 2013^45^ | Japan | 12 patients | Skull base tumor surgery | Pituitary tumor resection | Artis Zeego | - Navigation  - Postoperative control | Brainlab navigation system (Brainlab, Munich, Germany) |
| Peh 2020^46^ | Germany | 4 human cadavers | Spinal | Spine instrumentation | AlluraClarity | - Preoperative imaging and surgical planning  - Fluoroscopic guidance  - Navigation | ARSN |
| Balicki 2020^47^ | Sweden | 1 human cadaver | Spinal | Spine instrumentation | AlluraClarity FD20 | - Preoperative imaging and surgical planning  - Navigation  - Postoperative control | - Custom image-guided robotic arm for assistance in spine surgery - ARSN |
| Richter 2017^48^ | Germany | 10 spine models | Spinal | Spine instrumentation | Artis Zeego | - Preoperative imaging and surgical planning - Navigation | Syngo iGuide (Siemens Healthcasre, Forchheim, Germany |
| Schuetze 2019^49^ | Germany | 109 patients | Spinal | - Spine instrumentation - Other non-neurosurgical procedures | Artis Zeego | - Intraoperative imaging and confirmation - Navigation  - Postoperative control | Brainlab navigation system (Brainlab, Munich, Germany) |
| Schroeder 2022^50^ | Israel | 27 patients | Spinal | Spine instrumentation | Artis Zeego | - Preoperative imaging and surgical planning - Navigation - Postoperative control | Renaissance (Mazor Robotics) robotic guidance system |
| Elmi-Terander 2018^51^ | Sweden | 3 human cadavers | Spinal | Spine instrumentation | Allura Clarity FD20 | - Preoperative imaging and surgical planning - Navigation - Postoperative control | ARSN |
| Nachabe 2019^52^ | Netherlands | 3 phantoms | Spinal | n/a | AlluraClarity | Not mentioned | - |
| Nevzati 2017^53^ | Switzerland | 36 patients | Spinal | Spine instrumentation | AlluraXper FD20 | - Preoperative imaging and surgical planning  - Fluoroscopic guidance - Postoperative control | - |
| Burström 2020^54^ | Sweden | 4 human cadavers | Spinal | Spine instrumentation | AlluraClarity FD20 | - Preoperative imaging and surgical planning  - Navigation  - Postoperative control | - ARSN - Custom image-guided robotic arm for assistance in spine surgery |
| Pireau 2017^55^ | Belgium | 50 patients | Spinal | Spine instrumentation | Artis Zeego | - Preoperative imaging - Navigation  - Postoperative control | Syngo X workplace (Siemens Healthcare, Forchheim, Germany) |
| Pedicelli 2011^56^ | Italy | 950 procedures | Spinal | - Percutaneous treatment of discal herniation - Other non-neurosurgical procedures | Allura Xper FD20 | - Fluoroscopic guidance  - Intraoperative imaging - Postoperative control | - |
| Burström 2021^57^ | Sweden | 20 patients | Spinal | Spine instrumentation | Allura Xper FD20 | - Preoperative imaging and surgical planning - Navigation - Postoperative control | ARSN |
| Burström 2020^58^ | Sweden | - 4 human cadavers  - 20 patients | Spinal | Spine instrumentation | AlluraClarity Flexmove | - Preoperative imaging and surgical planning  - Navigation  - Postoperative control | ARSN |
| Kageyama 2017^59^ | Japan | 17 patients | Spinal | Spine instrumentation | Artis Zeego | - Surgical planning - Fluoroscopic guidance  - Postoperative control | - |
| Burström 2019^60^ | Sweden | 2 pig cadavers | Spinal | Spine instrumentation | AlluraClarity Flexmove | - Preoperative imaging and surgical planning - Navigation - Postoperative control | - ARSN - VR |
| Edstrom 2019^61^ | Sweden | 20 patients | Spinal | Spine instrumentation | Allura Clarity FlexMove | - Preoperative imaging and surgical planning - Navigation  - Postoperative control | ARSN |
| Burström 2019^62^ | Sweden | - 20 patients - 21 human cadavers | Spinal | N/a | Allura Clarity | Testing vertebral segmentation models for the purpose of neuronavigation in pedicle screw placement surgeries. | Artificial intelligence (AI) |
| Edström 2020^63^ | Sweden | 20 patients | Spinal | Spine instrumentation | Allura FlexMove | - Preoperative imaging and surgical planning - Navigation - Postoperative control | ARSN |
| Elmi-Terander 2019^64^ | Sweden | 20 patients | Spinal | Spine instrumentation | AlluraClarity Flexmove | - Preoperative imaging and surgical planning - Navigation  - Postoperative control | ARSN |
| Shin 2020^65^ | Japan | 23 patients | Spinal | Endoscopic spine surgery | Artis zeego | - Preoperative imaging and surgical planning - Navigation | Brainlab navigation system (Brainlab, Munich, Germany) |
| Bohoun 2018^66^ | Japan | 33 patients | Spinal | Spine instrumentation | AlluraClarity FD20 | - Intraoperative imaging  - Navigation - Postoperative control | Stealth Station (Medtronic Inc., Memphis, TN, USA |
| Fong 2020^67^ | Taiwan | 103 patients | Spinal | Spine instrumentation | Artis Zeego | - Preoperative imaging and surgical planning  - Navigation  - Postoperative control | Brainlab navigation system (Brainlab, Munich, Germany) |
| Elmi-Terander 2016^68^ | Sweden | 4 human cadavers | Spinal | Spine instrumentation | Allura Clarity FD20 | - Preoperative imaging and surgical planning  - Navigation | ARSN |
| Edström 2020^69^ | Sweden | 44 patients | Spinal | Spine instrumentation | Allura | - Preoperative imaging and surgical planning - Navigation - Postoperative control | ARSN |
| Tanikawa 2021^70^ | Japan | 58 patients | Spinal | Spine instrumentation | Artis Pheno | - Navigation - Postoperative control | Brainlab navigation system (Brainlab, Munich, Germany) |
| Fomekong 2017^71^ | Belgium | 66 patients | Spinal | Spine instrumentation | Artis Zeego | - Preoperative imaging and surgical planning  - Navigation - Postoperative control | -Brainlab navigation system (Brainlab, Munich, Germany) - Viper 2 System for PPS placement |
| Kaminski 2017^72^ | Belgium | 97 patients | Spinal | Spine instrumentation | Artis Zeego | - Fluoroscopic guidance  - Navigation  - Postoperative control | Syngo X workplace (Siemens Healthcare, Forchheim, Germany |
| Cewe 2021^73^ | Sweden | N/a | Spinal | n/a | AlluraClarity | No procedures were performed | - |
| Elmi-Terander 2020^74^ | Sweden | 40 patients | Spinal | Spine instrumentation | Allura Flexmove | - Preoperative imaging and surgical planning - Navigation - Postoperative control | ARSN |

Abbreviation: AVF = arteriovenous fistula, AVM = arteriovenous malformation, AI = artificial intelligence, ARSN = Augmented-Reality surgical navigation, N/a = not applicable.

Table B. Aims and conclusions of the included studies

| **Study ID** | **Aims and conclusions** |
| --- | --- |
| **Byval 2018** | Hybrid operating room provides the opportunity to combine endovascular and microsurgical methods, optimizing the treatment of complex vascular anomalies. Although it is expensive, it allows for intraoperative evaluation and confirmation, preventing revision surgery. |
| **Burström 2021** | Intraoperative CBCT was shown to be reliable in ruling out pedicle screw breaches. It can be used for breach detection and to assess the need for revision intraoperatively, possibly replacing routine postoperative CT scans |
| **Kienzler 2020** | Intraoperative endovascular TBO was shown to be a feasible, safe, and valuable method for the clipping of complex posterior circulation aneurysms. Intraoperative DSA and 3D-DSA were shown to be useful for assessing aneurysm occlusion and detecting residual aneurysms in a hybrid OR. |
| **Peh 2020** | The placement of MISS screws using ARSN was shown to be as accurate as the conventional fluoroscopy method. When the time needed for CBCT acquisition and planning was taken into account, the time for wire insertion was significantly longer for ARSN. |
| **Burström 2020** | By using optical video camera and adhesive skin makers for patient tracking, the ARSN system does not require registration or calibration and can easily be integrated in the surgical workflow without obstructing the surgical field. It is also highly accurate regardless of vertebral position. |
| **Schaller 2011** | The hybrid operating room could improve the workflow in treatment of aneurysmal subarachnoid hemorrhage as it allows the completion of all diagnostic and interventional activities in the same room, without the need to transfer the patient. |
| **Balicki 2020** | A robotic guidance system, integrated with a hybrid OR, was shown to provide a simple workflow and precise pedicle screw guidance with high technical and clinical accuracy. |
| **Fierstra 2020** | Vascular segmentation with 3D-iDSA, which is possible in hybrid operating rooms, provides an improved roadmap that corrects brain shift and improves the accuracy of neuronavigation. By intraoperatively updating the neuronavigation, valuable feedback about the location of vascular lesions can be provided. |
| **Richter 2017** | Laser guidance was shown to be feasible for pedicle screw placement. This new technique provided a pedicle screw accuracy comparable to that of the conventional technique, but it is believed that it could provide additional help for pedicle screw implantation even in challenging locations. Furthermore, the procedure time was significantly longer compared to the conventional method but could potentially be shortened with training and experience. |
| **Liao 2019** | Only a minority of procedures (20%) seem to benefit from operation in a hybrid operating room. While most of these procedures are only slightly enhanced by simultaneous high-resolution imaging, in some, such as combined open and endovascular procedures the use of a hybrid OR is crucial. Recognizing that combined endovascular and microsurgical techniques are key in the treatment of complex cerebrovascular diseases gives grounds for the utilization of hybrid ORs. Therefore, these novel environments should be regarded as a good alternative for cases that were previously considered impossible to treat. Ultimately, the use of the hybrid operating room, when indicated, depended on both its availability and the preference of the operating surgeon. |
| **Schuetze 2019** | Inside hybrid operating rooms, radiation dose is different for the different staff. Radiation dose of the surgeon was significantly higher than that of the nurse, however neither of them will exceed the yearly radiation limit if the surgeon wears apron and assuming they perform 300 surgeries a year. The use of navigation reduced radiation exposure without increasing the duration of operation for dorsal instrumentation of the thoracic spine. |
| **Iihara 2013** | Hybrid ORs allows the combination of endovascular and surgical procedures for 1-stage management of complex brachiocephalic and neurovascular lesions. |
| **Zhang 2020** | Hybrid-ORs can benefit from an intraoperative DSA system for accurate localization of shunts and confirmation of fistula obliteration, reducing the error obliteration rate. |
| **Neki 2020** | The minimally invasive Direct trans-sinus embolization (dTSE) procedure using a single burr hole for the treatment of isolated-type DAVFs was shown to be effective and to reduce contrast usage compared to the transvenous embolization method. Although dTSE led to a slightly reduced operation time and radiation exposure, the difference was not statistically significant. |
| **Zhang 2020** | The hybrid-OR could open new avenues for the removal of large and giant intracranial carotid-ophthalmic aneurysms. It led to a reduction in the rates of vessel stenosis, as well as remnant aneurysms. |
| **Mori 2013** | The use of DynaCT for intraoperative visualization during transsphenoidal pituitary tumor resection was described. This new technique can be used in conjunction with conventional transsphenoidal surgery to improve the rate of resection and decrease the injury to the cavernous sinus and internal carotid artery. |
| **Marbacher 2019** | Both ICGVA and intraoperative DSA are very useful for intraoperative confirmation of surgical results, supporting the idea that they are complementary rather than competitive. The gold standard regarding surgical outcome remains iDSA. |
| **Kato 2021** | Combined endovascular embolization and craniotomy in the hybrid operating room was shown to be an effective treatment option for refractory intracranial AVFs. This technique resulted in a good occlusion rate with a low complication rate. |
| **Shimada 2020** | Compared to intravenous videoangiography ICG videoangiography, intraarterial ICG videoangiography was more useful to detect feeders, nidus, and drainer and to assess the flow dynamics of the nidus in cerebral AVM surgery. |
| **Kageyama 2017** | The major benefits of a hybrid OR equipped with a multi-axis angiography unit, for spinal surgery, are the increased accuracy and safety as well as the reduced time required for confirmation of the Percutaneous Pedicle Screw |
| **Kim 2020** | Various neurosurgical procedures were performed safely and efficiently in the hybrid operating room. Combined endovascular and open surgery procedures that can be performed in hybrid operating rooms are less time consuming and safer than the conventional approach of independent surgery or endovascular surgery. |
| **Kim 2019** | Radiation exposure was shown to differ for the various roles in the hybrid OR, with the greatest exposure being for the operator. Reinforced protection, which consisted of an additional lead screen, significantly decreased the exposure, particularly for the operator. Interestingly, radiation exposure of radiologists increased with reinforced protection (although not significantly), suggesting that more attention should be paid to their location and movement during procedures. Effective doses of all personal were acceptable. |
| **Choi 2019** | The hybrid operating room allows for the combination of endovascular and open surgery, which provides new surgical strategies for the management of complex cerebrovascular disease. A hybrid operating room also allows for rapid switching between treatment strategies to overcome complications, increasing patient safety. |
| **Marbacher 2021** | During aneurysm treatment, both intraoperative and postoperative 3DSA were shown to be more sensitive than 2DSA in detecting clip remnants, particularly smaller ones. 3D-DSA is better than 2DSA for the visualization of small remnants independently of the number of clips or complexity of local angioarchitecture and thus would be better for studying the long-term risk of recurrence of completely clipped IA or small remnants. |
| **Kawamura 2017** | Intraluminal shunt for intraoperative shunt angiography could replace conventional DSA. The shunt provided a suturing scaffold that prevented the risk of postoperative stenosis. Intraluminal shunt and shunt angiography were shown to be effective for carotid artery near occlusion. |
| **Burström 2019** | Intraoperative augmented reality navigation was shown to be feasible for the placement of the pedicle screw. It provides intraoperative feedback on the deep anatomic structures, facilitating precise placement of the pedicle screws with no radiation exposure to the staff. |
| **Edstrom 2019** | Staff radiation exposure was lower than the values reported in the literature as well as the annual occupational dose limit recommended by the International Commission on Radiological Protection. Real-time feedback dosimeters lead to a decrease in radiation exposure during the last procedures performed, by allowing the personnel to optimize their positioning behind the lead shield in a way to reduce the dose. Correctly used lead shield spare the need for lead aprons which could be uncomfortable for the surgeons. Therefore, the use of a navigation system in the hybrid operating room along with real-time dosimeter feedback could reduce staff radiation exposure. In addition, patient exposure was relatively low. |
| **Burström 2019** | Automated neuronavigation technologies may enhance surgical workflow and accuracy in pedicle screw placement. |
| **Xin 2021** | The combined endovascular and surgical procedure in a hybrid operating room is an effective and safe treatment strategy for AVM. Interventional therapy is valuable in combined treatment as it helps achieve complete obliteration of the vascular lesions. |
| **Edström 2020** | The use of ARSN in the hybrid OR could increase surgical accuracy and patient safety while decreasing radiation exposure of the staff. The use of a navigation system did not add much to the operation time, which could potentially be further reduced by optimizing the workflow. |
| **Elmi-Terander 2019** | The use of ARSN navigation system for pedicle screw placement in a hybrid OR achieved a high accuracy with acceptable navigation time. |
| **Shin 2020** | Navigation-assisted endoscopic spine surgery was shown to be feasible in a hybrid operating room. The use of a navigation system was shown to be a safe and effective tool for intraoperative guidance. |
| **Fong 2018** | Intraoperative angiography techniques including 2D-DSA and 3D-RA integrated in the hybrid operating room can be safely used to achieve a better intraoperative assessment of surgical outcome in aneurysm surgeries. The use of 3D-RA may not be routinely required but is invaluable for complex aneurysms. |
| **Zheng 2013** | The hybrid operating room provided the opportunity to perform endovascular treatment and craniotomy clipping simultaneously during a single surgery, opening up new avenues for the treatment of complex intracranial aneurysms. |
| **Marbacher 2020** | Both intra- and postoperative DSA provided excellent image quality. The medical assessment by both modalities was also comparable. The results suggest that intraoperative DSA could possibly replace the need for postoperative DSA. |
| **Schroeder 2022** | The combination of the two robots, 3D C-arm (ArtisZeego) and the robotic- guidance platform Renaissance (Mazor Robotics) might improve surgical outcomes and increase patient safety and accuracy. |
| **Murayama 2013** | The hybrid operating room provides safer grounds for conducting combined endovascular and surgical interventions for the treatment of complex and risk-bearing neurovascular conditions. |
| **Elmi-Terander 2018** | Minimally invasive pedicle screw placement under the guidance of AR and intraoperative 3D imaging in a hybrid OR is accurate and efficient, with no need for fluoroscopy or X-ray imaging. |
| **Murayama 2010** | The robotic DSA system was shown to be very flexible, allowing switching between endovascular and open procedures without moving the patient. It provided a safe and precise treatment. The system could benefit of some improvements such as increased rotation velocity and a more OR friendly user interface. An interdisciplinary collaboration is needed for the integration of such system. |
| **Nachabe 2019** | CBCT imaging in a hybrid OR exposed the patient to lower radiation doses while still maintaining higher contrast to noise ratio and Hounsfield accuracy as compared to that provided by the O-arm. |
| **Bohoun 2018** | Spinal fusion surgery could be performed safely and accurately using the hybrid OR. Radiation exposure of the patient and the surgeon was shown to be under the limit recommended by the Japan association of Radiological Technologists and International commission on Radiological Protection, respectively. |
| **Murayama 2006** | Endovascular operating room provides a means of safe and effective neurosurgical practice. For optimal treatment, the neuro-endovascular team must be familiar with both open and endovascular procedures. |
| **Nevzati 2017** | Compared to the traditional post-operative CT scan, the accuracy of intraoperative CT (iCT) was 82%. iCT achieved high sensitivity and specificity in detecting severe violations and, therefore, could be a valuable tool for intraoperative evaluation of screw position. This would allow immediate correction preventing later revision surgery. |
| **Fong 2020** | The use of the navigation system was shown to be accurate and safe for the placement of pedicle screws. It decreased the number of fluoroscopic images taken, reducing radiation exposure of the surgical personnel. Although it resulted in increased operation time for the two-level surgeries, this could potentially be reduced by increased familiarity with the system and an optimized workflow. |
| **Goren 2020** | The use of 3D-RA and 2D-IOA was useful for the intraoperative assessment of aneurysm obliteration. 3D-RA proved to be superior to 2D-IOA in detecting residual aneurysm but did not add significant information for DAVF and AVM resections and direct bypasses. |
| **Elmi-Terander, 2016** | The use of an optical-based augmented reality navigation on a C-arm imaging system in the hybrid OR provided a higher pedicle screw accuracy compared to the free-hand technique. |
| **Burström 2020** | The use of a new robotic guidance system integrated into the hybrid operating room ARSN system is feasible and provides high technical and clinical accuracy for pedicle cannulation. |
| **Dammann 2017** | For the treatment of ruptured intracranial aneurysms, there was no statistically significant difference between hybrid OR and conventional treatment groups in terms of remnant frequency, clipping-related vascular insults, and functional outcomes. Nonetheless, patients in the hybrid operating room could benefit from simultaneous endovascular treatment if needed. |
| **Skyrman 2021** | The ARSN navigation system can accurately guide EVD insertion and cranial biopsy. It can potentially improve workflow of cranial biopsies in the surgical setting. |
| **Jiang 2019** | Multimodality in-situ recanalization in Hybrid Operating Room (MIRHOR) for symptomatic chronic internal carotid artery occlusions during a single session improved technical success and was associated with favorable clinical outcomes and a low rate of periprocedural complications. |
| **Edström 2020** | Compared to free-hand technique, the use of ARSN increased pedicle screw density without prolonging the operation time; however, this did not significantly impact the short-term correction rate. ARSN enabled the surgeon to place more pedicle screws possibly resulting in better constructs with long term advantages and decreased need for revision surgery. |
| **Jeon 2019** | Combined endovascular and surgical procedures can be performed simultaneously in the hybrid operating room, providing new and safe treatment plans for the management of complex cerebrovascular diseases. In a hybrid operating room, if the endovascular or surgical approach fails, the surgeon can immediately switch to another treatment strategy in an attempt to overcome the complications encountered. In addition, CBCT could be useful for intraoperative CT and frameless stereotaxic surgery without the need for pre-operative images. |
| **Grüter 2018** | Combined endovascular and microsurgical procedures in a hybrid OR is safe, effective and feasible for the radical treatment of low-grade AVMs. Interventional DSA allows the surgeon to adapt his microsurgical approach after intraoperative embolization and iDSA, improving surgical and radiological results. |
| **Kim 2020** | Using fluoroscopy and peel-away sheath for placement of ventriculoarterial shunt in a hybrid operating room could be a good alternative for the management of hydrocephalus in patients with shunt failure due to complications of the peritoneal cavity. |
| **Nossek 2017** | Intraoperative angiography is important in the treatment of spinal and neurovascular lesions. Both brachial and radial access can safely be obtained in a hybrid OR. Even though the prone, semi prone, and lateral positions constituted an angiographic challenge, operator experience and high-quality imaging technology in the hybrid OR enabled decent intraoperative visualization and postoperative control. |
| **Tsuei 2018** | For IPAP cases, rapid and aggressive conversion to neurosurgical rescue procedures in a hyrbid OR may improve patient outcome. For such cases, the hybrid OR allows rapid detection and management without the need to move the patient to a different facility. |
| **Pireau 2017** | The low-dose protocol yielded a higher accuracy of pedicle screw placement compared to the high-dose protocol, with a five-fold decrease in radiation exposure. Thus, low protocol reduces radiation exposure without compromising accuracy. It might be suggested to perform the control CBCT for the evaluation of the implant position using the low-dose protocol instead of the high-dose protocol. |
| **Park 2020** | Combination of IOA and ICGV affects clip adjustment decisions, as using IOA after ICGV increased the adjustment rate. IOA can confirm the safety of clipping status when the illuminating field is limited or when the interpretation of ICGV is vague. |
| **Song 2021** | The hybrid operation room was efficient for the treatment of cerebrovascular diseases. It could boost the surgeon's confidence and achieve favorable patient outcomes with a low rate of complications. It allowed for the combination of embolization and resection in one stage, which was shown to be a safe approach for the repair of low- and high-grade AVM. |
| **Durner 2021** | Although ICG videoangiography is as safe as intraoperative DSA, more available, less expensive and time-consuming, it cannot replace it in some cases, particularly when the field of view is restricted. |
| **Tanikawa 2021** | Using lower doses of radiation did not significantly increase the rate of pedicle screw violation. Violations were more likely in short patients and close to the upper instrumented vertebrae. |
| **Choi 2022** | Combined open and endovascular interventions, facilitated by hybrid OR, provide safe grounds to efficiently overcome unfavorable anatomy of the vertebral artery and treat complex posterior cerebrovascular diseases. |
| **Ashour 2016** | Modifications applied to the neuro-endovascular operating suite were described. The modified suite allows surgical access to the head while having the C-arm positioned in the lower body of the patient. This facilitates the transition between angiographic and open surgery positions, decreasing the procedure time and improving workflow efficiency. Hybrid operating room is believed to improve patient outcome and create opportunities for new treatment strategies by allowing the combination of surgical and endovascular techniques. |
| **Fomekong 2017** | The use of computer-assisted navigation based on io3D fluoroscopic images in a hybrid OR, allowed the accurate placement of pedicle screws while reducing the staff radiation exposure to undetectable doses. Furthermore, the use of a navigation system reduced the number of fluoroscopic acquisitions required, reducing the radiation exposure to the patient. |
| **Kobayashi 2012** | VPS placement under flat panel detector CT‑guided real‑time fluoroscopy was shown to be accurate and resulted in a lower misplacement rate compared to the conventional method. This was also associated with decreased early revision rate. |
| **Yu 2016** | Interventional embolization followed by craniotomy for AVM resection was performed safely in a hybrid operating room. |
| **Groter 2018** | Patients who underwent microsurgical-endovascular treatment of complex dAVFs in the hybrid showed a high rate of fistula obliteration immediately and at follow-up. Some temporary complications developed and were managed, but no long-term adverse effects developed. |
| **Wang 2020** | Although intraoperative angiography faces technical difficulties and complications when the patient is not in the supine position, IOA performed in a three-quarter prone position was shown to be safe through the femoral access. This approach is a useful tool for the treatment of high cervical and intracranial AVM / AVM. |
| **Irie 2008** | Although still novel, DynaCT can be implemented into the hybrid OR for image-based surgical planning and guidance. In comparison with conventional CBCT, DynaCT yielded lower quality images, however, scanning a wider area in a shorter time. Flat-panel detector-based, but not Image intensifier-based, DynaCT image quality was sufficient to identify small amounts of SAH or intracerebral hematoma. These technologies still require further improvements to achieve higher contrast resolution. |
| **Pedicelli 2011** | Although bearing lower contrast resolution and being more susceptible to motion artifacts, CBCT may act as a valid alternative to Multi-Slice CT for fluoroscopy-guided spinal procedures, with lower financial costs. |
| **Kaminski 2017** | The surgical technique and acquisition protocol affect the patient's radiation exposure: radiation exposure of the patient was shown to be 2 times higher in MIS compared to the conventional approach and the low-dose protocol reduced radiation exposure by three folds. Other factors that influence intraoperative radiation hazard are the patient's BMI and the operating time. These 4 factors explained 68% of the variance of radiation exposure in this study. Among them, acquisition protocol was the best predictor of DAP, followed by patient BMI, surgical technique, and finally operative time. |
| **Fandino 2013** | Hybrid OR provides the opportunity to combine endovascular and microsurgical procedures in a single session, a safe practice with cost-benefit advantages. |
| **Cewe 2021** | Occupational radiation exposure was lower with fixed CBCT compared to mobile CBCT, possibly due to the fact that hCBCT employs an automatic exposure control. The radiation exposure of the staff can be minimized during 3D image guided spine procedures by optimizing their positioning in the OR. Radiation protection shields can be used instead of lead aprons to effectively reduce whole body dose with improved comfort. |

**References**

1. Ashour, R., See, A. P., Dasenbrock, H. H., Khandelwal, P., Patel, N. J., Belcher, B., & Aziz-Sultan, M. A. (2016). Refinement of the Hybrid Neuroendovascular Operating Suite: Current and Future Applications. *World Neurosurgery*, *91*, 6–11. https://doi.org/10.1016/j.wneu.2016.03.055

2. Ashour, R., See, A. P., Dasenbrock, H. H., Khandelwal, P., Patel, N. J., Belcher, B., & Aziz-Sultan, M. A. (2016). Refinement of the Hybrid Neuroendovascular Operating Suite: Current and Future Applications. *World Neurosurgery*, *91*, 6–11. https://doi.org/10.1016/j.wneu.2016.03.055

3. Murayama, Y., Arakawa, H., Ishibashi, T., Kawamura, D., Ebara, M., Irie, K., Takao, H., Ikeuchi, S., Ogawa, T., Kato, M., Kajiwara, I., Nishimura, S., & Abe, T. (2013). Combined surgical and endovascular treatment of complex cerebrovascular diseases in the hybrid operating room. *Journal of NeuroInterventional Surgery*, *5*(5), 489–493. https://doi.org/10.1136/neurintsurg-2012-010382

4. Murayama, Y., Irie, K., Saguchi, T., Ishibashi, T., Ebara, M., Nagashima, H., Isoshima, A., Arakawa, H., Takao, H., Ohashi, H., Joki, T., Kato, M., Tani, S., Ikeuchi, S., & Abe, T. (2011). Robotic digital subtraction angiography systems within the hybrid operating room. *Neurosurgery*, *68*(5), 1427–1432. https://doi.org/10.1227/NEU.0b013e31820b4f1c

5. Schaller, K., Kotowski, M., Pereira, V., Rüfenacht, D., & Bijlenga, P. (2011). *From Intraoperative Angiography to Advanced Intraoperative Imaging: The Geneva Experience* (pp. 111–115). https://doi.org/10.1007/978-3-211-99651-5_18

6. Irie, K., Murayama, Y., Saguchi, T., Ishibashi, T., Ebara, M., Takao, H., & Abe, T. (2008). DynaCT soft-tissue visualization using an angiographic c-arm system: Initial clinical experience in the operating room. *Neurosurgery*, *62*(3 SUPPL. 1), 266–272; discussion 272. https://doi.org/10.1227/01.neu.0000317403.23713.92

7. Liao, C. H., Chen, W. H., Lee, C. H., Shen, S. C., & Tsuei, Y. S. (2019). Treating cerebrovascular diseases in hybrid operating room equipped with a robotic angiographic fluoroscopy system: level of necessity and 5-year experiences. *Acta Neurochirurgica*, *161*(3), 611–619. https://doi.org/10.1007/s00701-018-3769-4

8. Dammann, P., Jägersberg, M., Kulcsar, Z., Radovanovic, I., Schaller, K., & Bijlenga, P. (2017). Clipping of ruptured intracranial aneurysms in a hybrid room environment—a case-control study. *Acta Neurochirurgica*, *159*(7), 1291–1298. https://doi.org/10.1007/s00701-017-3212-2

9. Song, J., Li, P., Tian, Y., An, Q., Liu, Y., Yang, Z., Chen, L., Quan, K., Gu, Y., Ni, W., Zhu, W., & Mao, Y. (2021). One-Stage Treatment in a Hybrid Operation Room to Cure Brain Arteriovenous Malformation: A Single-Center Experience. *World Neurosurgery*, *147*, e85–e97. https://doi.org/10.1016/j.wneu.2020.11.123

10. Choi, E., Lee, J. Y., Cho, H. J., & Yoon, D. Y. (2022). Surgical exposure of the vertebral artery for endovascular access in a hybrid operating room. *Acta Neurochirurgica*, *164*(5), 1271–1280. https://doi.org/10.1007/s00701-022-05136-7

11. Yu, J. L., Guo, Y. B., Xu, B. F., Chen, X., & Xu, K. (2016). Onyx embolization and surgical removal as a treatment for hemorrhagic AVM in a hybrid operating room. *INTERNATIONAL JOURNAL OF CLINICAL AND EXPERIMENTAL MEDICINE*, *9*(11), 22494–22501.

12. Byval’tsev, V. A., Belykh, E. G., Kikuta, K. ichiro, & Stepanov, I. A. (2018). A Hybrid Neurosurgical Operating Room: Potentials in the Treatment of Arteriovenous Malformations of the Brain. *Biomedical Engineering*, *52*(1), 14–18. https://doi.org/10.1007/s10527-018-9772-5

13. Kienzler, J. C., Diepers, M., Marbacher, S., Remonda, L., & Fandino, J. (2020). Endovascular temporary balloon occlusion for microsurgical clipping of posterior circulation aneurysms. *Brain Sciences*, *10*(6). https://doi.org/10.3390/brainsci10060334

14. Fierstra, J., Anon, J., Mendelowitsch, I., Fandino, J., Diepers, M., Remonda, L., & Marbacher, S. (2020). Amended Intraoperative Neuronavigation: Three-Dimensional Vascular Roadmapping with Selective Rotational Digital Subtraction Angiography. *World Neurosurgery*, *135*, 183–187. https://doi.org/10.1016/j.wneu.2019.12.055

15. Iihara, K., Satow, T., Matsushige, T., Kataoka, H., Nakajima, N., Fukuda, K., Isozaki, M., Maruyama, D., Nakae, T., & Hashimoto, N. (2013). Hybrid operating room for the treatment of complex neurovascular and brachiocephalic lesions. *Journal of Stroke and Cerebrovascular Diseases*, *22*(8), E277–E285. https://doi.org/10.1016/j.jstrokecerebrovasdis.2012.07.014

16. Zhang, N., & Xin, W. Q. (2020). Application of hybrid operating rooms for treating spinal dural arteriovenous fistula. *World Journal of Clinical Cases*, *8*(6), 1056–1064. https://doi.org/10.12998/wjcc.v8.i6.1056

17. Neki, H., Yonezawa, A., Shibata, A., Tsukagoshi, E., Yamane, F., Ishihara, S., & Kohyama, S. (2020). A minimally invasive approach for the treatment of isolated type intracranial dural arteriovenous fistula in a neurosurgical hybrid operating room. *Interdisciplinary Neurosurgery: Advanced Techniques and Case Management*, *21*. https://doi.org/10.1016/j.inat.2020.100762

18. Zhang, N., & Xin, W. Q. (2020). Application of hybrid operating rooms for clipping large or giant intracranial carotid-ophthalmic aneurysms. *World Journal of Clinical Cases*, *8*(21), 5149–5158. https://doi.org/10.12998/wjcc.v8.i21.5149

19. Marbacher, S., Mendelowitsch, I., Grüter, B. E., Diepers, M., Remonda, L., & Fandino, J. (2019). Comparison of 3D intraoperative digital subtraction angiography and intraoperative indocyanine green video angiography during intracranial aneurysm surgery. *Journal of Neurosurgery*, *131*(1), 64–71. https://doi.org/10.3171/2018.1.JNS172253

20. Kato, N., Ishibashi, T., Maruyama, F., Otani, K., Kakizaki, S., Nagayama, G., Ikemura, A., Hataoka, S., Kan, I., Kodama, T., & Murayama, Y. (2021). Clinical outcomes of procedures combining endovascular embolization with a direct surgical approach in a hybrid operating room for the treatment of refractory dural arteriovenous fistulas. *Surgical Neurology International*, *12*, 439. https://doi.org/10.25259/SNI_486_2021

21. Shimada, K., Yamaguchi, T., Miyamoto, T., Sogabe, S., Korai, M., Okazaki, T., Kanematsu, Y., Satomi, J., Nagahiro, S., & Takagi, Y. (2021). Efficacy of intraarterial superselective indocyanine green videoangiography in cerebral arteriovenous malformation surgery in a hybrid operating room. *Journal of Neurosurgery*, *134*(5), 1544–1552. https://doi.org/10.3171/2020.3.JNS20319

22. Kim, T., Kwon, O. K., Ban, S. P., Kim, Y. D., & Won, Y. D. (2019). A Phantom Menace to Medical Personnel During Endovascular Treatment of Cerebral Aneurysms: Real-Time Measurement of Radiation Exposure During Procedures. *World Neurosurgery*, *125*, e289–e296. https://doi.org/10.1016/j.wneu.2019.01.063

23. Choi, E., Lee, J. Y., Jeon, H. J., Cho, B. M., & Yoon, D. Y. (2019). A hybrid operating room for combined surgical and endovascular procedures for cerebrovascular diseases: a clinical experience at a single centre. *British Journal of Neurosurgery*, *33*(5), 490–494. https://doi.org/10.1080/02688697.2019.1617403

24. Marbacher, S., & Steiger, H. J. (2021). Letter: Commentary: Value of 3-Dimensional Digital Subtraction Angiography for Detection and Classification of Intracranial Aneurysm Remnants after Clipping. *Operative Neurosurgery*, *21*(4), E406. https://doi.org/10.1093/ons/opab249

25. Kawamura, Y., Maruyama, D., Akagi, Y., & Iihara, K. (2017). Effective Intraluminal Shunt in Carotid Endarterectomy for Carotid Artery Near Occlusion: Technical Report. *World Neurosurgery*, *106*, 813–818. https://doi.org/10.1016/j.wneu.2017.07.078

26. Xin, C., Luo, W. ting, Zhao, W. yuan, Dong, L. xin, Xiong, Z. wei, Li, Z. wei, Zhang, J. jian, & Chen, J. cao. (2021). Combined Endovascular and Surgical Treatment for Brain Arteriovenous Malformations in Biplanar Hybrid Operating Room. *Current Medical Science*, *41*(4), 782–787. https://doi.org/10.1007/s11596-021-2398-y

27. Fong, Y. W., Hsu, S. K., Huang, C. T., Hsieh, C. T., Chen, M. H., Huang, J. S., Chang, C. J., & Su, I. C. (2018). Impact of Intraoperative 3-Dimensional Volume-Rendering Rotational Angiography on Clip Repositioning Rates in Aneurysmal Surgery. *World Neurosurgery*, *114*, e573–e580. https://doi.org/10.1016/j.wneu.2018.03.035

28. Zheng, S.-P., Sun, H., & You, C. (2013). Hybrid operating room: Combined operative and endovascular treatment for intracranial aneurysms. *Neurosurgery Quarterly*. https://doi.org/10.1097/WNQ.0000000000000012

29. Marbacher, S., Kienzler, J. C., Mendelowitsch, I., D’Alonzo, D., Andereggen, L., Diepers, M., Remonda, L., & Fandino, J. (2020). Comparison of intra- And postoperative 3-dimensional digital subtraction angiography in evaluation of the surgical result after intracranial aneurysm treatment. *Neurosurgery*, *87*(4), 689–696. https://doi.org/10.1093/neuros/nyz487

30. Murayama, Y., Saguchi, T., Ishibashi, T., Ebara, M., Takao, H., Irie, K., Ikeuchi, S., Onoue, H., Ogawa, T., & Abe, T. (2006). Endovascular operating suite: Future directions for treating neurovascular disease. *Journal of Neurosurgery*, *104*(6), 925–930. https://doi.org/10.3171/jns.2006.104.6.925

31. Goren, O., Bourdages, G., Schirmer, C. M., Weiner, G., Dalal, S. S., & Griessenauer, C. J. (2020). Intraoperative 3-Dimensional Rotational Angiography in Cerebrovascular Surgery: A Case Series. *World Neurosurgery*, *141*, e736–e742. https://doi.org/10.1016/j.wneu.2020.06.026

32. Jiang, W. J., Liu, A. F., Yu, W., Qiu, H. C., Zhang, Y. Q., Liu, F., Li, C., Wang, R., Zhao, Y. L., Lv, J., Li, T. X., Liu, C., Zhou, J., & Zhao, J. Z. (2019). Outcomes of Multimodality in situ Recanalization in Hybrid Operating Room (MIRHOR) for symptomatic chronic internal carotid artery occlusions. *Journal of NeuroInterventional Surgery*, *11*(8), 825–832. https://doi.org/10.1136/neurintsurg-2018-014384

33. Jeon, H. J., Lee, J. Y., Cho, B. M., Yoon, D. Y., & Oh, S. M. (2019). Four-year experience using an advanced interdisciplinary hybrid operating room : Potentials in treatment of cerebrovascular disease. *Journal of Korean Neurosurgical Society*, *62*(1), 35–45. https://doi.org/10.3340/jkns.2018.0203

34. Grüter, B. E., Mendelowitsch, I., Diepers, M., Remonda, L., Fandino, J., & Marbacher, S. (2018). Combined Endovascular and Microsurgical Treatment of Arteriovenous Malformations in the Hybrid Operating Room. *World Neurosurgery*, *117*, e204–e214. https://doi.org/10.1016/j.wneu.2018.05.241

35. Grüter, B. E., Strange, F., Burn, F., Remonda, L., Diepers, M., Fandino, J., & Marbacher, S. (2018). Hybrid Operating Room Settings for Treatment of Complex Dural Arteriovenous Fistulas. *World Neurosurgery*, *120*, e932–e939. https://doi.org/10.1016/j.wneu.2018.08.193

36. Nossek, E., Chalif, D. J., Buciuc, R., Gandras, E. J., Anderer, E. G., Insigna, S., Dehdashti, A. R., & Setton, A. (2017). Intraoperative angiography for arteriovenous malformation resection in the prone and lateral positions, using upper extremity arterial access. *Operative Neurosurgery*, *13*(3), 352–360. https://doi.org/10.1093/ons/opw034

37. Tsuei, Y. S., Liao, C. H., Lee, C. H., Liang, Y. J., Chen, W. H., & Yang, S. F. (2018). Intraprocedural arterial perforation during neuroendovascular therapy: Preliminary result of a dual-trained endovascular neurosurgeon in the neurosurgical hybrid operating room. *Journal of the Chinese Medical Association*, *81*(1), 31–36. https://doi.org/10.1016/j.jcma.2017.05.012

38. Park, J. H., Lee, J. Y., Jeon, H. J., Lim, B. C., Park, S. W., & Cho, B. M. (2020). Safety and completeness of using indocyanine green videoangiography combined with digital subtraction angiography for aneurysm surgery in a hybrid operating theater. *Neurosurgical Review*, *43*(4), 1163–1171. https://doi.org/10.1007/s10143-019-01141-0

39. Durner, G., Wahler, H., Braun, M., Kapapa, T., Wirtz, C. R., König, R., & Pala, A. (2021). The value of intraoperative angiography in the time of indocyanine green videoangiography in the treatment of cerebrovascular lesions: Efficacy, workflow, risk-benefit and cost analysis A prospective study. *Clinical Neurology and Neurosurgery*, *205*, 106628. https://doi.org/10.1016/j.clineuro.2021.106628

40. Wang, C., Hsu, S. K., Chang, C. J., Chen, M. H., Huang, C. T., Huang, J. S., & Su, I. C. (2020). Transfemoral Approach for Intraoperative Angiography in the Prone or Three-quarter Prone Position: A Revisited Protocol for Intracranial Arteriovenous Malformation and Fistula Surgery. *Clinical Neuroradiology*, *30*(2), 373–379. https://doi.org/10.1007/s00062-019-00783-3

41. Fandino, J., Taussky, P., Marbacher, S., Muroi, C., Diepers, M., Fathi, A. R., & Remonda, L. (2013). The concept of a hybrid operating room: Applications in cerebrovascular surgery. *Acta Neurochirurgica, Supplementum*, *115*, 113–117. https://doi.org/10.1007/978-3-7091-1192-5_24

42. Skyrman, S., Lai, M., Edström, E., Burström, G., Förander, P., Homan, R., Kor, F., Holthuizen, R., Hendriks, B. H. W., Persson, O., & Elmi-Terander, A. (2021). Augmented reality navigation for cranial biopsy and external ventricular drain insertion. *Neurosurgical Focus*, *51*(2), 1–7. https://doi.org/10.3171/2021.5.FOCUS20813

43. Kim, Y. H., Lee, S. W., Kim, D. H., Lee, C. H., Kim, C. H., Sung, S. K., Son, D. W., & Song, G. S. (2020). Case series of ventriculoatrial shunt placement in hybrid room: Reassessment of ventriculoatrial shunt. *Korean Journal of Neurotrauma*, *16*(2), 181–189. https://doi.org/10.13004/KJNT.2020.16.E45

44. Kobayashi, S., Ishikawa, T., Mutoh, T., Hikichi, K., & Suzuki, A. (2012). A novel technique for ventriculoperitoneal shunting by flat panel detector CT-guided real-time fluoroscopy. *Surgical Neurology International*, *3*(1), 119. https://doi.org/10.4103/2152-7806.102330

45. Mori, R., Joki, T., Matsuwaki, Y., Karagiozov, K., Murayama, Y., & Abe, T. (2013). Initial experience of real-time intraoperative C-arm computed-tomography- guided navigation surgery for pituitary tumors. *World Neurosurgery*, *79*(2), 319–326. https://doi.org/10.1016/j.wneu.2012.10.011

46. Peh, S., Chatterjea, A., Pfarr, J., Schäfer, J. P., Weuster, M., Klüter, T., Seekamp, A., & Lippross, S. (2020). Accuracy of augmented reality surgical navigation for minimally invasive pedicle screw insertion in the thoracic and lumbar spine with a new tracking device. *Spine Journal*, *20*(4), 629–637. https://doi.org/10.1016/j.spinee.2019.12.009

47. Balicki, M., Kyne, S., Toporek, G., Holthuizen, R., Homan, R., Popovic, A., Burström, G., Persson, O., Edström, E., Elmi-Terander, A., & Patriciu, A. (2020). Design and control of an image-guided robot for spine surgery in a hybrid OR. *International Journal of Medical Robotics and Computer Assisted Surgery*, *16*(4), e2108. https://doi.org/10.1002/rcs.2108

48. Richter, P. H., Gebhard, F., Salameh, M., Schuetze, K., & Kraus, M. (2017). Feasibility of laser-guided percutaneous pedicle screw placement in the lumbar spine using a hybrid-OR. *International Journal of Computer Assisted Radiology and Surgery*, *12*(5), 873–879. https://doi.org/10.1007/s11548-017-1529-1

49. Schuetze, K., Eickhoff, A., Dehner, C., Schultheiss, M., Gebhard, F., & Richter, P. H. (2019). Radiation exposure for the surgical team in a hybrid-operating room. *Journal of Robotic Surgery*, *13*(1), 91–98. https://doi.org/10.1007/s11701-018-0821-6

50. Schroeder, J. E., Houri, S., Weil, Y. A., Liebergall, M., Moshioff, R., & Kaplan, L. (2022). When giants talk; robotic dialog during thoracolumbar and sacral surgery. *BMC Surgery*, *22*(1), 125. https://doi.org/10.1186/s12893-022-01546-7

51. Elmi-Terander, A., Nachabe, R., Skulason, H., Pedersen, K., Söderman, M., Racadio, J., Babic, D., Gerdhem, P., & Edström, E. (2018). Feasibility and accuracy of thoracolumbar minimally invasive pedicle screw placement with augmented reality navigation technology. *Spine*, *43*(14), 1018–1023. https://doi.org/10.1097/BRS.0000000000002502

52. Nachabe, R., Strauss, K., Schueler, B., & Bydon, M. (2019). Radiation dose and image quality comparison during spine surgery with two different, intraoperative 3D imaging navigation systems. *Journal of Applied Clinical Medical Physics*, *20*(2), 136–145. https://doi.org/10.1002/acm2.12534

53. Nevzati, E., Fandino, J., Schatlo, B., Heimberg, M., Marbacher, S., Remonda, L., & Fathi, A. R. (2017). Validation and accuracy of intraoperative CT scan using the Philips AlluraXper FD20 angiography suite for assessment of spinal instrumentation. *British Journal of Neurosurgery*, *31*(6), 741–746. https://doi.org/10.1080/02688697.2017.1297764

54. Burström, G., Balicki, M., Patriciu, A., Kyne, S., Popovic, A., Holthuizen, R., Homan, R., Skulason, H., Persson, O., Edström, E., & Elmi-Terander, A. (2020). Feasibility and accuracy of a robotic guidance system for navigated spine surgery in a hybrid operating room: a cadaver study. *Scientific Reports*, *10*(1), 7522. https://doi.org/10.1038/s41598-020-64462-x

55. Pireau, N., Cordemans, V., Banse, X., Irda, N., Lichtherte, S., & Kaminski, L. (2017). Radiation dose reduction in thoracic and lumbar spine instrumentation using navigation based on an intraoperative cone beam CT imaging system: a prospective randomized clinical trial. *European Spine Journal*, *26*(11), 2818–2827. https://doi.org/10.1007/s00586-017-5229-x

56. Pedicelli, A., Verdolotti, T., Pompucci, A., Desiderio, F., D’Argento, F., Colosimo, C., & Bonomo, L. (2011). Interventional spinal procedures guided and controlled by a 3D rotational angiographic unit. *Skeletal Radiology*, *40*(12), 1595–1601. https://doi.org/10.1007/s00256-011-1282-4

57. Burström, G., Cewe, P., Charalampidis, A., Nachabe, R., Söderman, M., Gerdhem, P., Elmi-Terander, A., & Edström, E. (2021). Intraoperative cone beam computed tomography is as reliable as conventional computed tomography for identification of pedicle screw breach in thoracolumbar spine surgery. *European Radiology*, *31*(4), 2349–2356. https://doi.org/10.1007/s00330-020-07315-5

58. Burström, G., Balicki, M., Patriciu, A., Kyne, S., Popovic, A., Holthuizen, R., Homan, R., Skulason, H., Persson, O., Edström, E., & Elmi-Terander, A. (2020). Feasibility and accuracy of a robotic guidance system for navigated spine surgery in a hybrid operating room: a cadaver study. *Scientific Reports*, *10*(1), 7522. https://doi.org/10.1038/s41598-020-64462-x

59. Kageyama, H., Yoshimura, S., Uchida, K., & Iida, T. (2017). Advantages and disadvantages of multi-axis intraoperative angiography unit for percutaneous pedicle screw placement in the lumbar spine. *Neurologia Medico-Chirurgica*, *57*(9), 481–488. https://doi.org/10.2176/nmc.oa.2017-0059

60. Burström, G., Nachabe, R., Persson, O., Edström, E., & Elmi Terander, A. (2019). Augmented and Virtual Reality Instrument Tracking for Minimally Invasive Spine Surgery: A Feasibility and Accuracy Study. *Spine*, *44*(15), 1097–1104. https://doi.org/10.1097/BRS.0000000000003006

61. Edström, E., Burström, G., Nachabe, R., Gerdhem, P., & Terander, A. E. (2020). A novel augmented-reality-based surgical navigation system for spine surgery in a hybrid operating room: Design, workflow, and clinical applications. *Operative Neurosurgery*, *18*(5), 496–502. https://doi.org/10.1093/ons/opz236

62. Burström, G., Buerger, C., Hoppenbrouwers, J., Nachabe, R., Lorenz, C., Babic, D., Homan, R., Racadio, J. M., Grass, M., Persson, O., Edström, E., & Elmi Terander, A. (2019). Machine learning for automated 3-dimensional segmentation of the spine and suggested placement of pedicle screws based on intraoperative cone-beam computer tomography. *Journal of Neurosurgery: Spine*, *31*(1), 147–154. https://doi.org/10.3171/2018.12.SPINE181397

63. Burström, G., Nachabe, R., Homan, R., Hoppenbrouwers, J., Holthuizen, R., Persson, O., Edström, E., & Elmi-Terander, A. (2020). Frameless Patient Tracking With Adhesive Optical Skin Markers for Augmented Reality Surgical Navigation in Spine Surgery. *Spine*, *45*(22), 1598–1604. https://doi.org/10.1097/BRS.0000000000003628

64. Elmi-Terander, A., Burström, G., Nachabe, R., Skulason, H., Pedersen, K., Fagerlund, M., Ståhl, F., Charalampidis, A., Söderman, M., Holmin, S., Babic, D., Jenniskens, I., Edström, E., & Gerdhem, P. (2019). Pedicle Screw Placement Using Augmented Reality Surgical Navigation with Intraoperative 3D Imaging: A First In-Human Prospective Cohort Study. *Spine*, *44*(7), 517–525. https://doi.org/10.1097/BRS.0000000000002876

65. Shin, Y., Sunada, H., Shiraishi, Y., Hosokawa, M., Koh, Y., Tei, R., Aketa, S., Motoyama, Y., Yonezawa, T., & Nakase, H. (2020). Navigation-assisted full-endoscopic spine surgery: a technical note. *Journal of Spine Surgery (Hong Kong)*, *6*(2), 513–520.

66. Bohoun, C. A., Naito, K., Yamagata, T., Tamrakar, S., Ohata, K., & Takami, T. (2019). Safety and accuracy of spinal instrumentation surgery in a hybrid operating room with an intraoperative cone-beam computed tomography. *Neurosurgical Review*, *42*(2), 417–426. https://doi.org/10.1007/s10143-018-0977-6

67. Fong, Y. W., Su, I. C., Hsieh, C. T., Huang, C. T., & Chang, C. J. (2020). Accuracy and safety of pedicle screws implantation using Zeego and Brainlab navigation system in hybrid operation room. *Formosan Journal of Surgery*, *53*(2), 48–54. https://doi.org/10.4103/fjs.fjs_65_19

68. Elmi-Terander, A., Skulason, H., Soderman, M., Racadio, J., Homan, R., Babic, D., van der Vaart, N., & Nachabe, R. (2016). Surgical navigation technology based on augmented reality and integrated 3D intraoperative imaging a spine cadaveric feasibility and accuracy study. *Spine*, *41*(21), E1303–E1311. https://doi.org/10.1097/BRS.0000000000001830

69. Edström, E., Burström, G., Persson, O., Charalampidis, A., Nachabe, R., Gerdhem, P., & Elmi-Terander, A. (2020). Does Augmented Reality Navigation Increase Pedicle Screw Density Compared to Free-Hand Technique in Deformity Surgery? Single Surgeon Case Series of 44 Patients. *Spine*, *45*(17), E1085–E1090. https://doi.org/10.1097/BRS.0000000000003518

70. Tanikawa, Y., Oba, H., Fujii, M., Ikegami, S., Uehara, M., Mimura, T., Miyagawa, J., Hatakenaka, T., Kuraishi, S., Takizawa, T., Munakata, R., Kamanaka, T., Miyaoka, Y., Michihiko, K., & Takahashi, J. (2022). Intraoperative Cone Beam CT in Hybrid Operation Room for Pediatric Scoliosis Patients. *Spine*, *47*(12), E507–E513. https://doi.org/10.1097/brs.0000000000004226

71. Fomekong, E., Safi, S. E., & Raftopoulos, C. (2017). Spine Navigation Based on 3-Dimensional Robotic Fluoroscopy for Accurate Percutaneous Pedicle Screw Placement: A Prospective Study of 66 Consecutive Cases. *World Neurosurgery*, *108*, 76–83. https://doi.org/10.1016/j.wneu.2017.08.149

72. Kaminski, L., Cordemans, V., Cartiaux, O., & van Cauter, M. (2017). Radiation exposure to the patients in thoracic and lumbar spine fusion using a new intraoperative cone-beam computed tomography imaging technique: a preliminary study. *European Spine Journal*, *26*(11), 2811–2817. https://doi.org/10.1007/s00586-017-4968-z

73. Cewe, P., Vorbau, R., Omar, A., Elmi-Terander, A., & Edström, E. (2021). Radiation distribution in a hybrid operating room, utilizing different X-ray imaging systems: investigations to minimize occupational exposure. *Journal of NeuroInterventional Surgery*, neurintsurg-2021-018220. https://doi.org/10.1136/neurintsurg-2021-018220

74. Elmi-Terander, A., Burström, G., Nachabé, R., Fagerlund, M., Ståhl, F., Charalampidis, A., Edström, E., & Gerdhem, P. (2020). Augmented reality navigation with intraoperative 3D imaging vs fluoroscopy-assisted free-hand surgery for spine fixation surgery: a matched-control study comparing accuracy. *Scientific Reports*, *10*(1), 707. https://doi.org/10.1038/s41598-020-57693-5

Table C. Latest and insightful case reports on the use of hybrid-OR in neurosurgery

| **Study ID** | **Condition** | **Use** | **Case report and interesting insight** |
| --- | --- | --- | --- |
| (Okada et al. 2022) | Trauma | Simultaneous open/endovascular treatment | Decompressive craniotomy for severe traumatic brain injury was performed simultaneously as endovascular embolization for intraperitoneal injured arteries, without any operative complications. |
| (Liu et al. 2022) | AVM | Intraoperative imaging | Endovascular imaging capabilities of the hybrid-OR were used during open surgery of a complex AVM case to evaluate the presence of residuals. |
| (Wang et al. 2022) | AVM | Intraoperative imaging | Endovascular imaging capabilities of the hybrid-OR were used during open surgery of a complex case of a coexistence of both primitive trigeminal artery and AVM to evaluate the presence of residuals. |
| (Yamaguchi et al. 2022) | Dural arteriovenous fistula | Simultaneous open/endovascular treatment | Transcortical venous coil embolization as well as open hematoma removal were performed in a single session for the management of a dural arteriovenous fistula presenting with massive hematoma. |
| (Chen et al. 2022) | Spinal aneurysms | Conversion from endovascular to open approach | Open surgery and clipping was performed after failure of coil embolization in the management of a ruptured spinal artery aneurysm. |
| (Xie et al. 2022) | AVM | Simultaneous open/endovascular treatment | Successful treatment of the concomitant occurrence of AVMs and intracranial aneurysms with associated subarachnoid hemorrhage and frontal lobe hematoma using a hybrid open and endovascular approach. |
| (Shimada et al. 2021) | Hemangio-blastoma | Intraoperative imaging | Intra-arterial indocyanine green (ICG) from a catheter in the vertebral artery was used to guide the open surgical removal of a brainstem hemangioblastoma, which, according to the authors, was more useful than intravenous ICG videoangiography. |
| (Yang et al. 2022) | AVM | Simultaneous open/endovascular treatment | Awake, combined open and endovascular surgery for the management of eloquent brain AVMs with concomitant intracranial aneurysms. Multimodal treatment approach facilitated by the hybrid OR provide a safe option in the management of complex cerebrovascular pathologies. |
| (SATO et al. 2021) | Dural arteriovenous fistula | Simultaneous open/endovascular treatment | Simultaneous treatment of a dural arteriovenous fistula by coil embolization via a contralateral superficial middle cerebral vein and middle cerebral artery aneurysm by clipping in a hybrid OR. |
| (Kato et al. 2021) | Moyamoya disease | Intraoperative imaging | Extracranial-intracranial bypass surgery was performed in a hybrid OR. Bypass patency was verified intraoperatively using ICG videoangiography and Doppler ultrasonography. |
| (Kawano et al. 2020) | Trauma | Simultaneous open/endovascular treatment | A case of penetrating transorbital cavernous sinus injury treated by craniotomy as well as simultaneous balloon catheterization of the internal carotid artery to prevent bleeding during removal of the foreign object. |
| (McClendon et al. 2020) | Spine deformity | Intraoperative imaging and surgical planning | A case of spinal deformity repair in a hybrid OR. The use of both augmented reality and intraoperative imaging capability provided by the hybrid OR helped in visualization of proper screw trajectory, and in identification of correct screw placement and rod fixation. |
| (Hosokawa et al. 2019) | Intracranial aneurysms | Conversion from open to endovascular approach | Treatment of an unruptured posterior communicating artery aneurysm using, at first, clipping which was seamlessly reversed into endovascular coiling after ventricular tachycardia was encountered during the craniotomy. |
| (Ogiwara et al. 2019) | Basilar invagination | Intraoperative imaging | The hOR, allowed for the extent of bone resection and anatomic orientation to be confirmed intraoperatively, during endoscopic endonasal odontoidectomy. |
| (Gao et al. 2019) | Spinal arteriovenous fistula | Simultaneous open/endovascular treatment | Hybrid open and endovascular management of a filum terminale arteriovenous fistula in the lower sacral region, without any operative complications. |
| (Konakondla et al. 2018) | Intracranial aneurysms | Simultaneous open/endovascular treatment | Mechanical thrombectomy after intraoperative discovery of a distal large vessel occlusion during open surgical clipping of a posterior communicating artery aneurysm in a hybrid OR. |

**References:**

Chen F, Lu W, Lian B, Kang D, Dai L (2022) Isolated Cervical Ruptured Radiculomedullary Artery Aneurysm Predominantly Presenting as Supratentorial Subarachnoid Hemorrhage: Case Report and Review of the Literature. Brain Sci 12. https://doi.org/10.3390/BRAINSCI12050519

Gao P, Li X, Li G (2019) Retrograde Cannulation of the Draining Vein for Embolization of Filum Terminale Arteriovenous Fistula in the Lower Sacral Region. World Neurosurg 130:254–258. https://doi.org/10.1016/J.WNEU.2019.06.236

Hosokawa Y, Fukuda H, Fukui N, Hamada F, Yatabe T, Aoyama B, Hoashi Y, Higuchi S, Ueba Y, Furushima T, Ueba T (2019) [Failed Surgical Clipping of a Posterior Communicating Artery Aneurysm with Oculomotor Nerve Palsy due to Ventricular Tachycardia:Validity of Endovascular Coiling in the Hybrid Operating Room]. No Shinkei Geka 47:877–882. https://doi.org/10.11477/MF.1436204039

Kato N, Kan I, Abe Y, Otani K, Narikiyo M, Nagayama G, Nishimura K, Mori R, Kodama T, Ishibashi T, Murayama Y (2021) Visualization of extracranial-intracranial bypass in moyamoya patients using intraoperative three-dimensional digital subtraction angiography with intravenous contrast injection and robotic C-arm: patient series. Journal of neurosurgery Case lessons 1. https://doi.org/10.3171/CASE2057

Kawano T, Ohta H, Kawano T, Futami M, Ryu S, Sugimoto T, Akiba D, Yokogami K, Takeshima H (2020) [A Case of Transorbital Penetrating Cavernous Sinus Injury by a Bamboo Stick Treated by Craniotomy in the Hybrid Operating Room]. No Shinkei Geka 48:607–613. https://doi.org/10.11477/MF.1436204239

Konakondla S, Griessenauer CJ, Fong RP, Goren O, Schirmer CM (2018) Zero-Delay Mechanical Thrombectomy for Distal Large Vessel Occlusion Detected on Intraoperative Angiogram After Microsurgical Clipping of a Posterior Communicating Artery Aneurysm: Value of Hybrid Operating Room. World Neurosurg 119:278–281. https://doi.org/10.1016/J.WNEU.2018.08.088

Liu DD, Kurland DB, Ali A, Golfinos JG, Nossek E, Riina HA (2022) Pial brainstem artery arteriovenous malformation with flow-related intracanalicular aneurysm masquerading as vestibular schwannoma: illustrative case. Journal of neurosurgery Case lessons 4. https://doi.org/10.3171/CASE22208

McClendon J, Almekkawi AK, Abi-Aad KR, Maiti T (2020) Use of Pheno Room, Augmented Reality, and 3-Rod Technique for 3-Dimensional Correction of Adolescent Idiopathic Scoliosis. World Neurosurg 137:291. https://doi.org/10.1016/J.WNEU.2020.01.094

Ogiwara T, Miyaoka Y, Nakamura T, Tsukada K, Yamazaki D, Ito K, Hanaoka Y, Koyama J ichi, Horiuchi T, Hongo K (2019) Endoscopic Endonasal Odontoidectomy in the Hybrid Operating Room. World Neurosurg 131:137–140. https://doi.org/10.1016/J.WNEU.2019.07.197

Okada K, Tanei T, Kato T, Naito T, Koketsu Y, Ito R, Hirayama K, Hasegawa T (2022) Achieving good neurological outcome by combining decompressive craniectomy for acute subdural hematoma and transarterial embolization of intraperitoneal injured arteries for multiple severe trauma: a case report. Nagoya J Med Sci 84:640–647. https://doi.org/10.18999/NAGJMS.84.3.640

SATO S, KOKUBO Y, KAWANAMI K, ITAGAKI H, YAMADA Y, MATSUMOTO Y, SONODA Y (2021) Hybrid Surgical and Endovascular Approach via the Contralateral Superficial Middle Cerebral Vein to Occlude Cavernous Sinus Dural Arteriovenous Fistula in a Hybrid Operating Room: A Case Report. NMC Case Rep J 8:137–142. https://doi.org/10.2176/NMCCRJ.CR.2020-0070

Shimada K, YAMAMOTO Y, MIYAMOTO T, SOGABE S, FUJIHARA T, NAKAJIMA K, MIZOBUCHI Y, KANEMATSU Y, TAKAGI Y (2021) Efficacy of Intra-arterial Indocyanine Green Videoangiography in Hemangioblastoma Surgery: A Case Report. NMC Case Rep J 8:295–300. https://doi.org/10.2176/NMCCRJ.CR.2020-0281

Wang L, Li J, Li Z, Chai S, Chen J, Xiong N, Yang B (2022) Hybrid surgery for coexistence of cerebral arteriovenous malformation and primitive trigeminal artery: A case report and literature review. Front Surg 9. https://doi.org/10.3389/FSURG.2022.888558

Xie F, Huang L, Ye Y, Hao J, Lv J, Richard SA (2022) Hybrid operation for arteriovenous malformations with associated multiple intracranial aneurysms and subarachnoid hemorrhage: Case report. Medicine 101:E28944. https://doi.org/10.1097/MD.0000000000028944

Yamaguchi I, Kanematsu Y, Shimada K, Yamamoto N, Miyake K, Miyamoto T, Sogabe S, Shikata E, Ishihara M, Yamamoto Y, Kuroda K, Takagi Y (2022) Single-session hematoma removal and transcortical venous approach for coil embolization of an isolated transverse-sigmoid sinus dural arteriovenous fistula in a hybrid operating room: illustrative case. Journal of neurosurgery Case lessons 3. https://doi.org/10.3171/CASE2267

Yang Z, Deng M, Liu Y, Song J (2022) How I do it? A multimodality-guided awake hybrid operation for a language-area brain arteriovenous malformation and multiple intracranial aneurysms. Acta Neurochir (Wien) 164:1297–1301. https://doi.org/10.1007/S00701-021-05093-7
