# Supplementary material for: The use of hybrid operating rooms in neurosurgery, advantages, disadvantages, and future perspectives: a systematic review
Source: Acta Neurochir (Wien). 2023 Aug 16;165(9):2343–58. doi: 10.1007/s00701-023-05756-7 (PMC10477240; doi:10.1007/s00701-023-05756-7)
Supplement: Supplementary file 5 — Supplementary file5 (DOCX 111 KB) [file 701_2023_5756_MOESM5_ESM.docx]

**Risk of Bias assessment**

**Supplementary table 3.1.** Risk of bias assessment of observational studies according to the Newcastle-Ottawa scale.

|  | **Selection** | | | | **Comparability** | **Exposure** | | |  |
| --- | --- | --- | --- | --- | --- | --- | --- | --- | --- |
| **Title** | **Representativeness of the exposed cohort** | **Selection of the non-exposed cohort** | **Ascertainment of exposure** | **Demonstration that outcome of interest was not present at start of study** | **Comparability of cohorts on the basis of the design or analysis** | **Assessment of outcome** | **Was follow-up long enough for outcomes to occur** | **Adequacy of follow up of cohorts** | **Score** |
| A Hybrid Neurosurgical Operating Room: Potentials in the Treatment of Arteriovenous Malformations of the Brain | 0 | 1 | 1 | 1 | 1 | 1 | 1 | 1 | 5 |
| Intraoperative cone beam computed tomography is as reliable as conventional computed tomography for identification of pedicle screw breach in thoracolumbar spine surgery. | 1 | 1 | 1 | 1 | 1 | 1 | 1 | 1 | 8 |
| Endovascular Temporary Balloon Occlusion for Microsurgical Clipping of Posterior Circulation Aneurysms. | 0 | 1 | 1 | 1 | 1 | 1 | 1 | 1 | 5 |
| Frameless Patient Tracking With Adhesive Optical Skin Markers for Augmented Reality Surgical Navigation in Spine Surgery. | 1 | 1 | 1 | 1 | 1 | 1 | 1 | 1 | 6 |
| From Intraoperative Angiography to Advanced Intraoperative Imaging: The Geneva Experience | 1 | 1 | 1 | 1 | 1 | 1 | 1 | 1 | 6 |
| Amended Intraoperative Neuronavigation: Three-Dimensional Vascular Roadmapping with Selective Rotational Digital Subtraction Angiography | 0 | 1 | 1 | 1 | 1 | 1 | 1 | 1 | 5 |
| Treating cerebrovascular diseases in hybrid operating room equipped with a robotic angiographic fluoroscopy system: level of necessity and 5-year experiences. | 1 | 1 | 1 | 1 | 1 | 1 | 1 | 1 | 6 |
| Radiation exposure for the surgical team in a hybrid-operating room | 1 | 1 | 1 | 1 | 2 | 1 | 1 | 1 | 8 |
| Hybrid Operating Room for the Treatment of Complex Neurovascular and Brachiocephalic Lesions | 0 | 1 | 1 | 1 | 1 | 1 | 1 | 1 | 5 |
| Application of hybrid operating rooms for treating spinal dural arteriovenous fistula. | 0 | 1 | 1 | 1 | 1 | 1 | 1 | 1 | 5 |
| A minimally invasive approach for the treatment of isolated type intracranial dural arteriovenous fistula in a neurosurgical hybrid operating room | 0 | 1 | 1 | 1 | 1 | 1 | 1 | 1 | 5 |
| Application of hybrid operating rooms for clipping large or giant intracranial carotid-ophthalmic aneurysms. | 0 | 1 | 1 | 1 | 1 | 1 | 1 | 1 | 5 |
| Initial experience of real-time intraoperative C-arm computed-tomography-guided navigation surgery for pituitary tumors.. | 0 | 1 | 1 | 1 | 1 | 1 | 1 | 1 | 5 |
| Comparison of 3D intraoperative digital subtraction angiography and intraoperative indocyanine green video angiography during intracranial aneurysm surgery | 1 | 1 | 1 | 1 | 1 | 1 | 1 | 1 | 6 |
| Clinical outcomes of procedures combining endovascular embolization with a direct surgical approach in a hybrid operating room for the treatment of refractory dural arteriovenous fistulas. | 0 | 1 | 1 | 1 | 1 | 1 | 1 | 1 | 5 |
| Efficacy of intraarterial superselective indocyanine green videoangiography in cerebral arteriovenous malformation surgery in a hybrid operating room. | 0 | 1 | 1 | 1 | 1 | 1 | 1 | 1 | 5 |
| Advantages and Disadvantages of Multi-axis Intraoperative Angiography Unit for Percutaneous Pedicle Screw Placement in the Lumbar Spine | 0 | 1 | 1 | 1 | 1 | 1 | 1 | 1 | 5 |
| The experience of surgery and endovascular procedure of cerebrovascular disease in the hybrid operating room; Multi-axis robotic C-arm DSA system. | 1 | 1 | 1 | 1 | 1 | 1 | 1 | 1 | 6 |
| A Phantom Menace to Medical Personnel During Endovascular Treatment of Cerebral Aneurysms: Real-Time Measurement of Radiation Exposure During Procedures | 0 | 1 | 1 | 1 | 2 | 1 | 1 | 1 | 7 |
| A hybrid operating room for combined surgical and endovascular procedures for cerebrovascular diseases: a clinical experience at a single centre | 1 | 1 | 1 | 1 | 1 | 1 | 1 | 1 | 6 |
| Value of 3-Dimensional Digital Subtraction Angiography for Detection and Classification of Intracranial Aneurysm Remnants After Clipping | 1 | 1 | 1 | 1 | 1 | 1 | 1 | 1 | 8 |
| Effective Intraluminal Shunt in Carotid Endarterectomy for Carotid Artery Near Occlusion: Technical Report. | 0 | 1 | 1 | 1 | 1 | 1 | 1 | 1 | 5 |
| Augmented Reality Surgical Navigation in Spine Surgery to Minimize Staff Radiation Exposure | 1 | 1 | 1 | 1 | 2 | 1 | 1 | 1 | 8 |
| Combined Endovascular and Surgical Treatment for Brain Arteriovenous Malformations in Biplanar Hybrid Operating Room | 1 | 1 | 1 | 1 | 1 | 1 | 1 | 1 | 6 |
| A Novel Augmented-Reality-Based Surgical Navigation System for Spine Surgery in a Hybrid Operating Room: Design, Workflow, and Clinical Applications | 1 | 1 | 1 | 1 | 1 | 1 | 1 | 1 | 6 |
| Pedicle Screw Placement Using Augmented Reality Surgical Navigation With Intraoperative 3D Imaging: A First In-Human Prospective Cohort Study. | 1 | 1 | 1 | 1 | 1 | 1 | 1 | 1 | 6 |
| Navigation-assisted full-endoscopic spine surgery: a technical note. | 1 | 1 | 1 | 1 | 1 | 1 | 1 | 1 | 6 |
| Impact of Intraoperative 3-Dimensional Volume-Rendering Rotational Angiography on Clip Repositioning Rates in Aneurysmal Surgery. | 1 | 1 | 1 | 1 | 1 | 1 | 1 | 1 | 6 |
| Hybrid operating room: Combined operative and endovascular treatment for intracranial aneurysms | 1 | 1 | 1 | 1 | 1 | 1 | 1 | 1 | 6 |
| Comparison of Intra- and Postoperative 3-Dimensional Digital Subtraction Angiography in Evaluation of the Surgical Result After Intracranial Aneurysm Treatment. | 1 | 1 | 1 | 1 | 1 | 1 | 1 | 1 | 8 |
| When giants talk; robotic dialog during thoracolumbar and sacral surgery | 1 | 1 | 1 | 1 | 1 | 1 | 1 | 1 | 6 |
| Combined surgical and endovascular treatment of complex cerebrovascular diseases in the hybrid operating room. | 1 | 1 | 1 | 1 | 1 | 1 | 1 | 1 | 6 |
| Robotic digital subtraction angiography systems within the hybrid operating room | 0 | 1 | 1 | 1 | 1 | 1 | 1 | 1 | 5 |
| Radiation dose and image quality comparison during spine surgery with two different, intraoperative 3D imaging navigation systems. | 0 | 1 | 0 | 0 | 0 | 1 | 1 | 1 | 3 |
| Safety and accuracy of spinal instrumentation surgery in a hybrid operating room with an intraoperative cone-beam computed tomography | 1 | 1 | 1 | 1 | 1 | 1 | 1 | 1 | 6 |
| Endovascular operating suite: future directions for treating neurovascular disease. | 1 | 1 | 1 | 1 | 1 | 1 | 1 | 1 | 6 |
| Validation and accuracy of intraoperative CT scan using the Philips AlluraXper FD20 angiography suite for assessment of spinal instrumentation | 1 | 1 | 1 | 1 | 1 | 1 | 1 | 1 | 6 |
| Intraoperative 3-Dimensional Rotational Angiography in Cerebrovascular Surgery: A Case Series. | 1 | 1 | 1 | 1 | 1 | 1 | 1 | 1 | 6 |
| Outcomes of Multimodality In situ Recanalization in Hybrid Operating Room (MIRHOR) for symptomatic chronic internal carotid artery occlusions. | 1 | 1 | 1 | 1 | 1 | 1 | 1 | 1 | 6 |
| Four-Year Experience Using an Advanced Interdisciplinary Hybrid Operating Room : Potentials in Treatment of Cerebrovascular Disease. | 1 | 1 | 1 | 1 | 1 | 1 | 1 | 1 | 6 |
| Combined Endovascular and Microsurgical Treatment of Arteriovenous Malformations in the Hybrid Operating Room | 1 | 1 | 1 | 1 | 1 | 1 | 1 | 1 | 6 |
| Case Series of Ventriculoatrial Shunt placement in Hybrid Room: Reassessment of Ventriculoatrial Shunt. | 0 | 1 | 1 | 1 | 1 | 1 | 1 | 1 | 5 |
| Intraoperative Angiography for Arteriovenous Malformation Resection in the Prone and Lateral Positions, Using Upper Extremity Arterial Access. | 0 | 1 | 1 | 1 | 1 | 1 | 1 | 1 | 5 |
| Intraprocedural arterial perforation during neuroendovascular therapy: Preliminary result of a dual-trained endovascular neurosurgeon in the neurosurgical hybrid operating room | 0 | 1 | 1 | 1 | 1 | 1 | 1 | 1 | 5 |
| Safety and completeness of using indocyanine green videoangiography combined with digital subtraction angiography for aneurysm surgery in a hybrid operating theater. | 1 | 1 | 1 | 1 | 1 | 1 | 1 | 1 | 6 |
| One-Stage Treatment in a Hybrid Operation Room to Cure Brain Arteriovenous Malformation: A Single-Center Experience. | 1 | 1 | 1 | 1 | 1 | 1 | 1 | 1 | 6 |
| The value of intraoperative angiography in the time of indocyanine green videoangiography in the treatment of cerebrovascular lesions: Efficacy, workflow, risk-benefit and cost analysis A prospective study | 1 | 1 | 1 | 1 | 1 | 1 | 1 | 1 | 6 |
| Intraoperative cone beam CT in hybrid operation room for pediatric scoliosis patients: Comparison of pedicle screw violation rate at normal and low radiation doses | 1 | 1 | 1 | 1 | 1 | 1 | 1 | 1 | 6 |
| Surgical exposure of the vertebral artery for endovascular access in a hybrid operating room. | 0 | 1 | 1 | 1 | 1 | 1 | 1 | 1 | 5 |
| Refinement of the Hybrid Neuroendovascular Operating Suite: Current and Future Applications | 0 | 1 | 1 | 1 | 1 | 1 | 1 | 1 | 5 |
| Spine Navigation Based on 3-Dimensional Robotic Fluoroscopy for Accurate Percutaneous Pedicle Screw Placement: A Prospective Study of 66 Consecutive Cases. | 1 | 1 | 1 | 1 | 1 | 1 | 1 | 1 | 6 |
| A novel technique for ventriculoperitoneal shunting by flat panel detector CT‑guided real‑time fluoroscopy | 1 | 1 | 1 | 1 | 1 | 1 | 1 | 1 | 6 |
| Onyx embolization and surgical removal as a treatment for hemorrhagic AVM in a hybrid operating room | 0 | 1 | 1 | 1 | 1 | 1 | 1 | 1 | 5 |
| Hybrid Operating Room Settings for Treatment of Complex Dural Arteriovenous Fistulas | 0 | 1 | 1 | 1 | 1 | 1 | 1 | 1 | 5 |
| Transfemoral Approach for Intraoperative Angiography in the Prone or Three-quarter Prone Position A Revisited Protocol for Intracranial Arteriovenous Malformation and Fistula Surgery |  | 1 | 1 | 1 | 1 | 1 | 1 | 1 | 5 |
| Dynact soft-tissue visualization using an angiographic C-arm system: initial clinical experience in the operating room. | 1 | 1 | 1 | 1 | 1 | 1 | 1 | 1 | 6 |
| Interventional spinal procedures guided and controlled by a 3D rotational angiographic unit | 1 | 1 | 1 | 1 | 1 | 1 | 1 | 1 | 6 |
| Radiation exposure to the patients in thoracic and lumbar spine fusion using a new intraoperative cone-beam computed tomography imaging technique: a preliminary study | 1 | 1 | 1 | 1 | 1 | 1 | 1 | 1 | 6 |
| The concept of a hybrid operating room: applications in cerebrovascular surgery. | 1 | 1 | 1 | 1 | 1 | 1 | 1 | 1 | 6 |
| Radiation distribution in a hybrid operating room, utilizing different X-ray imaging systems: investigations to minimize occupational exposure | 0 | 1 | 0 | 0 | 1 | 1 | 1 | 1 | 3 |
| Accuracy of augmented reality surgical navigation for minimally invasive pedicle screw insertion in the thoracic and lumbar spine with a new tracking device | 0 | 1 | 1 | 1 | 2 | 1 | 1 | 1 | 8 |
| Design and control of an image-guided robot for spine surgery in a hybrid OR | 0 | 0 | 1 | 1 | 0 | 1 | 1 | 1 | 5 |
| Augmented and Virtual Reality Instrument Tracking for Minimally Invasive Spine Surgery: A Feasibility and Accuracy Study. | 0 | 0 | 1 | 1 | 0 | 1 | 1 | 1 | 5 |
| Feasibility and Accuracy of Thoracolumbar Minimally Invasive Pedicle Screw Placement With Augmented Reality Navigation Technology | 0 | 0 | 1 | 1 | 0 | 1 | 1 | 1 | 5 |
| Feasibility and accuracy of a robotic guidance system for navigated spine surgery in a hybrid operating room: a cadaver study. | 0 | 0 | 1 | 1 | 0 | 1 | 1 | 1 | 5 |
| Feasibility of laser-guided percutaneous pedicle screw placement in the lumbar spine using a hybrid-OR | 0 | 1 | 1 | 1 | 2 | 1 | 1 | 1 | 8 |
| Machine learning for automated 3-dimensional segmentation of the spine and suggested placement of pedicle screws based on intraoperative cone-beam computer tomography | 0 | 0 | 1 | 1 | 0 | 1 | 1 | 1 | 5 |
| Accuracy and safety of pedicle screws implantation using Zeego and Brainlab navigation system in hybrid operation room | 1 | 1 | 1 | 1 | 2 | 1 | 1 | 1 | 9 |
| Surgical Navigation Technology Based on Augmented Reality and Integrated 3D Intraoperative Imaging: A Spine Cadaveric Feasibility and Accuracy Study | 0 | 0 | 1 | 1 | 2 | 1 | 1 | 1 | 7 |
| Augmented reality navigation for cranial biopsy and external ventricular drain insertion | 0 | 0 | 1 | 1 | 0 | 1 | 1 | 1 | 5 |
| Does Augmented Reality Navigation Increase Pedicle Screw Density Compared to Free-Hand Technique in Deformity Surgery? Single Surgeon Case Series of 44 Patients | 1 | 1 | 1 | 1 | 2 | 1 | 1 | 1 | 9 |

**Supplementary table 3.2.** Risk of bias assessment of case-control studies using the Newcastle-Ottawa scale.

|  | **Selection** | | | | **Comparability** | **Exposure** | |  |
| --- | --- | --- | --- | --- | --- | --- | --- | --- |
| **Title** | **Is the case definition adequate?** | **Representativeness of the cases** | **Selection of Controls** | **Definition of Controls** | **Comparability of cases and controls on the basis of the design or analysis** | **Ascertainment of exposure** | **Same method of ascertainment for cases and controls** | **Total score** |
| Clipping of ruptured intracranial aneurysms in a hybrid room environment-a case-control study. | 1 | 1 | 1 | 1 | 2 | 1 | 1 | 8 |
| Augmented reality navigation with intraoperative 3D imaging vs fuoroscopy-assisted free-hand surgery for spine fxation surgery: a matched-control study comparing accuracy | 1 | 1 | 1 | 1 | 2 | 1 | 1 | 8 |
